# Sex Differences Revealed in a Mouse CFA Inflammation Model with Macrophage Targeted Nanotheranostics

**DOI:** 10.7150/thno.41309

**Published:** 2020-01-01

**Authors:** Lu Liu, Huseyin Karagoz, Michele Herneisey, Fatih Zor, Takaaki Komatsu, Shannon Loftus, Bratislav M. Janjic, Vijay S. Gorantla, Jelena M. Janjic

**Affiliations:** 1 Graduate School of Pharmaceutical Sciences, School of Pharmacy, Duquesne University, Pittsburgh, PA, USA.; 2 Chronic Pain Research Consortium, Duquesne University, Pittsburgh, PA, US.; 3 Department of Surgery, Institute of Regenerative Medicine, Wake Forest University School of Medicine, Winston-Salem, North Carolina, United States.; 4 Department of Pharmacology, Daiichi University of Pharmacy, Fukuoka, Japan.; 5 NRG Oncology Foundation Inc., University of Pittsburgh, Pittsburgh PA, United States.

**Keywords:** Pain nanomedicine, macrophages, sex differences, inflammatory pain, nanotheranostic.

## Abstract

Monocyte derived macrophages (MDMs) infiltrate sites of infection or injury and upregulate cyclooxygenase-2 (COX-2), an enzyme that stimulates prostaglandin-E2 (PgE2). Nanotheranostics combine therapeutic and diagnostic agents into a single nanosystem. In previous studies, we demonstrated that a nanotheranostic strategy, based on theranostic nanoemulsions (NE) loaded with a COX-2 inhibitor (celecoxib, CXB) and equipped with near-infrared fluorescent (NIRF) reporters, can specifically target circulating monocytes and MDMs. The anti-inflammatory and anti-nociceptive effects of such cell-specific COX-2 inhibition lasted several days following Complete Freund's Adjuvant (CFA) or nerve injury in male mice. The overall goal of this study was to investigate the extended (up to 40 days) impact of MDM-targeted COX-2 inhibition and any sex-based differences in treatment response; both of which remain unknown. Our study also evaluates the feasibility and efficacy of a preclinical nanotheranostic strategy for mechanistic investigation of the impact of such sex differences on clinical outcomes.

**Methods**: CFA was administered into the right hind paws of male and female mice. All mice received a single intravenous dose of NIRF labeled CXB loaded NE twelve hours prior to CFA injection. *In vivo* whole body NIRF imaging and mechanical hypersensitivity assays were performed sequentially and *ex vivo* NIRF imaging and immunohistopathology of foot pad tissues were performed at the end point of 40 days.

**Results**: Targeted COX-2 inhibition of MDMs in male and female mice successfully improved mechanical hypersensitivity after CFA injury. However, we observed distinct sex-specific differences in the intensity or longevity of the nociceptive responses. In males, a single dose of CXB-NE administered via tail vein injection produced significant improved mechanical hypersensitivity for 32 days as compared to the drug free NE (DF-NE) (untreated) control group. In females, CXB-NE produced similar, though less prominent and shorter-lived effects, lasting up to 11 days. NIRF imaging confirmed that CXB-NE can be detected up to day 40 in the CFA injected foot pad tissues of both sexes. There were distinct signal distribution trends between males and females, suggesting differences in macrophage infiltration dynamics between the sexes. This may also relate to differences in macrophage turnover rate between the sexes, a possibility that requires further investigation in this model.

**Conclusions**: For the first time, this study provides unique insight into MDM dynamics and the early as well as longer-term targeted effects and efficacy of a clinically translatable nanotheranostic agent on MDM mediated inflammation. Our data supports the potential of nanotheranostics as presented in elucidating the kinetics, dynamics and sex-based differences in the adaptive or innate immune responses to inflammatory triggers. Taken together, our study findings lead us closer to true personalized, sex-specific pain nanomedicine for a wide range of inflammatory diseases.

## Introduction

Monocyte-derived macrophages (MDMs) and other immune cells infiltrate sites of injury and produce numerous pro-inflammatory mediators (chemokines, cytokines, prostaglandins) that can sensitize neurons and induce pain 1. Macrophages, as drivers of inflammation, are implicated in many diseases that lead to chronic pain, from rheumatoid arthritis (RA) 2, 3, osteoarthritis (OA)^4^, and inflammatory bowel disease (IBD) 5 to trauma 6. In RA and OA, MDMs upregulate cytokine production and trigger T cell proliferation, which results in joint destruction and pain sensitization 3, 7.

An enzyme of particular interest in macrophage-driven inflammation and associated pain is cyclooxygenase-2 (COX-2), the primary enzyme driving the production of prostaglandin-E2 (PgE2) 8-10. In fact, macrophages expressing high levels of COX-2 have been shown to exacerbate the inflammation driven pathology in osteoarthritis 11. Nonsteroidal anti-inflammatory drugs (NSAIDs) have been the standard of care alternatives to opioids to treat pain and inflammation in RA, OA, and many other diseases 12. NSAIDs such as celecoxib (CXB) are potent inhibitors of COX-2 in immune cells (such as macrophages), which are critical drivers of the innate or adaptive responses to inflammation.

The importance of COX-2 in macrophage mediated inflammatory pain is supported by studies using macrophage COX-2 knockout mice. These studies showed reduced hyperalgesia post-CFA injection compared to wild type mice and reduced response to pretreatment with celecoxib, a COX-2 inhibitor 13. COX-2 knockout mice also fail to develop collagen-induced arthritis (CIA) compared to equally susceptible wild type and COX-1 knockout mice 14. Recent studies have shown that sex differences exist in COX-2 activity. Female COX-2 knockout mice exhibited reduced edema compared to wild type mice when subjected to complete Freund's adjuvant (CFA) injection, while male COX-2 knockout mice showed no significant reduction in edema 15. Sex differences are also seen in PgE2 production; macrophages from female mice subjected to trauma produced significantly more PgE2 compared to male mice experiencing the same trauma 16. Additionally, there are sex differences in macrophage activity, including higher activation and phagocytic activity in females 6.

These studies and our earlier work 10, 17 confirm that the macrophage enzyme COX-2 is a critical mediator of pain in inflammatory disease, making it an attractive therapeutic target. Nevertheless, the mechanisms underlying sex differences in clinical outcomes after COX-2 inhibition 18 are still poorly understood. We posit that the primary reason for this lies in variability in target cell effects following systemic dosing (oral or parenteral) of the drug in experimental models. One approach to this problem is delivery of NSAIDs directly to the immune cell mediators of inflammation, such as macrophages, using nanotheranostics. Nanotheranostics provide temporally and spatially controlled drug delivery to macrophages for prolonged intracellular drug exposure and simultaneous, longitudinal imaging of these cells to monitor for drug efficacy and potentially toxicity.

Here, we propose a novel nanotheranostic approach that could help provide preliminary insights into mechanistic elucidation of sex-intrinsic differences in macrophage migration dynamics and trafficking patterns to sites of inflammation, which can lead to advances in sex-specific, personalized pain nanomedicine. Our group has recently developed such a nanotheranostic approach, where COX-2 inhibitors were directly delivered to macrophages for both control and imaging of inflammation in multiple animal models using complex fluorescently labeled perfluorocarbon (PFC) nanoemulsions (NEs) as nanotheranostic formulations 17, 19, 20. Macrophages as nanotheranostic targets have been investigated recently in rodent models of inflammatory diseases and cancer 21-23. In contrast to these studies, presented work for the first time utilizes nanotheranostics in the context of identifying sex differences in inflammatory diseases by directly targeting macrophages. Furthermore, we also show that nanotheranostic formulations as presented can provide extended pain relief, bringing us closer to future personalized pain nanomedicine.

Nanotheranostics internalized by MDMs provide a unique cell specific signature for *in vivo* tracking using near-infrared fluorescence (NIRF), positron emission tomography (PET), or magnetic resonance imaging (MRI). NEs are small-size (<500 nm) oil-in-water emulsion droplets that are ideally suited for nanotheranostics, due to their surface-area-to-volume ratio that allows for effective drug loading, sustained release, and the ability to functionalize the surface with targeting ligands and imaging moieties (targeting agents, metal chelates, dyes, etc.) 24. Therefore theranostic NEs can serve as unique tools for studying the effects of NSAIDs on macrophage function *in vivo* and *ex vivo*. In the presented work, we used our established COX-2 inhibiting macrophage targeting nanotheranostic technology 17, 19 to investigate the effects of extended COX-2 inhibition in mouse models of inflammatory pain. Extensive analyses were performed in both males and females.

Presented study builds upon our earlier findings which demonstrated that NIRF-labeled celecoxib NEs (CXB-NEs) injected 12 hours prior to inflammatory insult are taken up by MDMs and found to associate specifically with COX-2-expressing macrophages in inflamed tissues 19. Furthermore, COX-2 inhibition in macrophages via CXB-NE caused changes in macrophage infiltration patterns as compared to drug-free NE (DF-NE) treated animals 19. In an experimental neuronal injury model, we also found that CXB-NE treatment decreased macrophage infiltration at the site of injury and caused dramatic reduction in mechanical hypersensitivity 17. In these studies, only male animals, mice 19 and rats^17^, were investigated, and we monitored NE-labeled macrophages for a limited time (4 days) post-injury and treatment.

To the best of our knowledge, this is the first study to show that a single dose of CXB delivered as an anti-inflammatory nanotheranostic formulation directly to macrophages can provide extended pain relief as well as allow elucidation of sex differences in pain behavior and inflammatory pain responses.

## Materials and Methods

Animals, male and female C57BL/6 mice (2-3 month old), strain code: 027 were purchased from Charles River Laboratories for imaging studies performed at Duquesne University under an approved IACUC protocol. Pathogen-free adult male and female C57BL/6 mice (CLEA, Tokyo, Japan) were used in the von Frey behavioral study. The experiments were performed with the approval of the committee of Animal Experiments in Daiichi College of Pharmaceutical Sciences, and complied with the recommendation of the International Association for the Study of Pain 25. The mice (18-25g) were maintained in a controlled 12 hour light-dark cycle with food and water ad libitum. Animals were used only once. CXB-NE and DF-NE carrying a NIRF reporter such as DiR'; DiIC18(7) (1,1'-Dioctadecyl-3,3,3',3'-Tetramethylindotricarbocyanine Iodide) from Invitrogen P12731 were manufactured following earlier established protocols 17, 26. Complete Freund's Adjuvant (CFA), Inject®, Thermo Scientific Product No. 77140.

### Preparation of NEs

The NEs used in this study (celecoxib loaded, CXB-NE and drug-free, DF-NE) were produced following earlier reported protocols in Janjic et al 17, 19. Briefly, NEs were prepared by incorporating the near-infrared fluorescent dye, DiR, DiIC18(7) (1,1'-Dioctadecyl-3,3,3',3'-Tetramethylindotricarbocyanine Iodide) (5 mM) with/without celecoxib (2.5 mg/ml) into a hydrocarbon oil. After adding perfluorocarbon and surfactants, the mixture was processed on a microfluidizer (Microfluidics, M110S) following earlier established methods 17.

### Colloidal characterization

Dynamic light scattering (Zetasizer Nano ZS, Malvern Instruments, UK) was used to measure droplet size and zeta potential at 25 °C. NE was dispersed in deionized water at a 1:80 dilution. Size and zeta potential were recorded on the same sample in a cuvette or standard zeta potential cell, respectively. Each sample was analyzed three times with at least 12 runs each. Example measurements for DF-NE and CXB-NE size distribution and zeta potential are shown in Figure S1A-D.

### Enzyme-Linked Immunosorbent Assay

RAW 264.7 murine macrophages (ATCC) were plated at 300,000 cells/2ml/well in 6 well plates for 24 hours. Cells were then exposed to the indicated concentrations of NE or drug dispersed in full media for 24 hours. The cells were then washed and exposed to serum-free media with or without 500 ng/ml LPS. After 18 hours, the cell culture supernatant was collected and centrifuged at 2000 rpm 4°C for two minutes to remove any cell debris. PgE2 was measured in these samples using the Prostaglandin E2 ELISA Kit - Monoclonal (Cayman Chemical, Ann Arbor USA) according to the manufacturer's instructions. CXB-NE inhibition of COX-2 in macrophages is shown in Figure S2A-B.

### Mechanical Hypersensitivity Assessments

Groups of male and female mice (n=5), weighing 18-25g, were treated with 200μl of CXB-NE, DF-NE or free drug solution (CXB dissolved in DMSO and saline mixture at 437.6µM) via tail vein injection. Twelve hours later each mouse received 50μl of CFA via intraplantar [i.pl] injection into the footpad of the right hind paw. The achieved dose for CXB was 25µg/injection, or approximately 1 mg/kg. Von Frey filaments were used to measure withdrawal threshold to mechanical stimulation to the plantar surface of each hind paw up to day 40 post CFA induction at distinct time points: days 0, 1, 3, 6, 11, 12, 18, 24, 32 and 40. 27. Briefly, treated and control mice were placed individually in a plastic cage (10 cm×10 cm×14 cm) with a wire mesh bottom. After mice had adapted to the testing environment for 60 min, a von Frey filament with 0.4 g bending force was pressed perpendicularly against the mid-plantar surface of the hind paw from below the mesh floor and held for 3-5 s with the filament slightly buckled. Lifting of the paw was recorded as a positive response. Stimulation of the same intensity (0.4 g filament) was applied to the point of bending ten times to the plantar surface of the hind paw of each mouse at intervals of 5 s. Behavioral testing was performed between 10 am and 4 pm. Testing was performed for two days before the start of the experiment to acclimatize the mice to testing procedures. Investigators were blinded to treatment interventions during pain behavior tests. All data analyses and statistical evaluations were performed by an independent operator.

### Near-Infrared Fluorescence Imaging

NIRF whole body imaging (WBI) was conducted in male and female mice with group size n=6. Animals were treated via tail vein injection with average dose of 200μl of NIRF labeled CXB-NE or DF-NE 12h prior to 50μl i.pl CFA injection into the footpad of the right hind paw. Whole body NIRF images were acquired at day 1, 3, 6, 9, 12, 18, 24, 32, and 40 post CFA injection on Pearl^®^ Trilogy Small Animal Imaging System. WBI imaging parameters were: 800nm channel resolution 255 µm; Focus offset No. 2 28-31. Images were analyzed and signal quantified using software Image Studio Lite. A rectangular box (area size 960) was placed on the back/shoulder region of each animal across the study, we assigned this selected dark shape as background: signal 0.000. Region of interest (ROIs) were drawn around each CFA-induced footpad and its contralateral footpad as control from toe to heel. Then, we recorded the signals from each footpad as our raw data (example shown in Figure S5). The animal was anesthetized with 1.5-2.5 % isoflurane/oxygen gas mixture inhalation. Then, the animal was placed on the pre-warmed to 37°C imaging chamber bed at prone position. We anchored the animal's face on the inhale nuzzle where they will continuously receive anesthesia. The animal's hind footpads were placed on each side of the tail, and the palm of hind paws were facing up. This allows the camera to acquire a clean shot of a picture representing the accumulation of NEs' NIRF signal of the inflamed footpad (hind right) and of the control footpad (hind left).

### Euthanasia and Organ distribution

The mice were euthanized under anesthesia immediately after the final imaging time point with a 0.5ml intraperitoneal injection of euthasol (pentobarbital sodium and phenytoin sodium solution, Virbac AH, Inc., Fort Worth, TX). The animals were immediately perfused with 20ml of PBS followed by 20ml of 4% paraformaldehyde in 1X PBS solution, administered into the left ventricle of the heart, resulting in whole body fixation. We dissected select internal organs and quantified fluorescence in each organ by drawing regions of interest (ROI) around each organ and normalizing the total fluorescence to the organ weight 19. In the biodistribution study (the *ex vivo* imaging), all male animals were evaluated using the Odyssey^®^CLx imaging system. The imaging parameters were: 800nm channel Intensity at 0.5; Focus 1.0. All the female animals were evaluated using the Pearl^®^ Trilogy Small Animal Imaging System. The imaging parameters were: 800nm channel Resolution 85 µm; Focus offset No. 2.

### Immunofluorescence and Histopathology Analyses

Male and female mice were sacrificed after 40 days post CFA injection, and both of the right and left paws were harvested. Tissues were prepared for histology with hematoxylin and eosin (H&E) and immunostaining. The excised tissue and organ samples were fixed in 4% PFA solution, embedded into paraffin, and cut into 10 μm sections using microtome (Leica, Buffalo Grove, IL). The sections were stained with H&E according to standard protocols. For immunostaining; non-specific binding was blocked with Dako serum free protein for 15 min at room temperature. Rat anti-mouse CD68 (Bio-Rad, Hercules, CA) (1/200 dilution in DPBS/0.1% BSA/0.05% Tween-20) and goat anti-mouse COX-2 antibodies (Novus, Centennial, CO) (at 1/50 dilution in PBS/0.1% BSA/0.05% Tween-20) were added overnight at 4 °C followed by a secondary antibody, anti-rat-Alexa 488 and anti-goat- Alexa 594 (Invitrogen, Grand Island, NY) (1/300 dilution in PBS/0.1% BSA/0.05% Tween-20) for 1 h at RT. After washing in DPBS/0.05% Tween-20, coverslips were mounted using Diamond anti-fading medium (Invitrogen, Grand Island, NY). Immunofluorescence staining was monitored using an Olympus BX63 fluorescence microscope with a multispectral camera by CRI (Perkin Elmer) and Nuance software.

### Statistical Analyses

Imaging and behavior tests data was analyzed and plotted using Graph Pad Prism 8 software. Data represents the mean ± SD, n = 5-6. Differences in treatment effects between groups for pain behavior analyses were determined using two-way ANOVA and Tukey's multiple comparison analyses between treatments (CXB-NE) and controls (DF-NE, free drug CXB, no treatment - CFA alone and saline alone). Statistical significance was determined using the Holm-Sidak method, with alpha=0.05. Each treatment was compared to controls at each time point was analyzed individually, without assuming a consistent standard deviation across groups over time. The statistical analyses results are provided in the Supplemental Information (Table S1 for males, Table S2 for females).

## Results

### CXB-NE not free-drug inhibits COX-2 in macrophages and provides long term pain control in male and female mice

Macrophage targeted NEs (CXB-NE) are designed for extended COX-2 inhibition resulting in prolonged suppression of PgE2 release from macrophages at the site of injury leading to mechanical hypersensitivity reduction 17. Due to the major contribution of COX-2 in macrophages in CFA induced pain, we hypothesized that CXB-NE can induce prolonged pain relief using the same strategy in mice. To test this hypothesis, 12 hours prior to CFA injection, groups of male and female mice were treated with a single dose of NIRF labeled CXB-NE, drug free NE (DF-NE) or free celecoxib (CXB) solution, and followed for 40 days using von Frey behavior assay. CXB-NE, DF-NE or free drug (CXB) solution were administered via tail vein injection in both males and females. Overall, the CXB dose was kept at 1mg/kg across all groups receiving drug treatment (CXB-NE or free drug) and the volume of injections (NE or free drug solution) was kept constant across treatment groups. In all behavior analyses the statistical significance was determined using two-way ANOVA with Tukey's multiple comparison methods, where withdrawals were compared for each treatment at each time point of follow up for both males and females. All pain behavior studies were performed using blinded operators to the treatment (CXB-NE or DF-NE).

**Figure 1:**
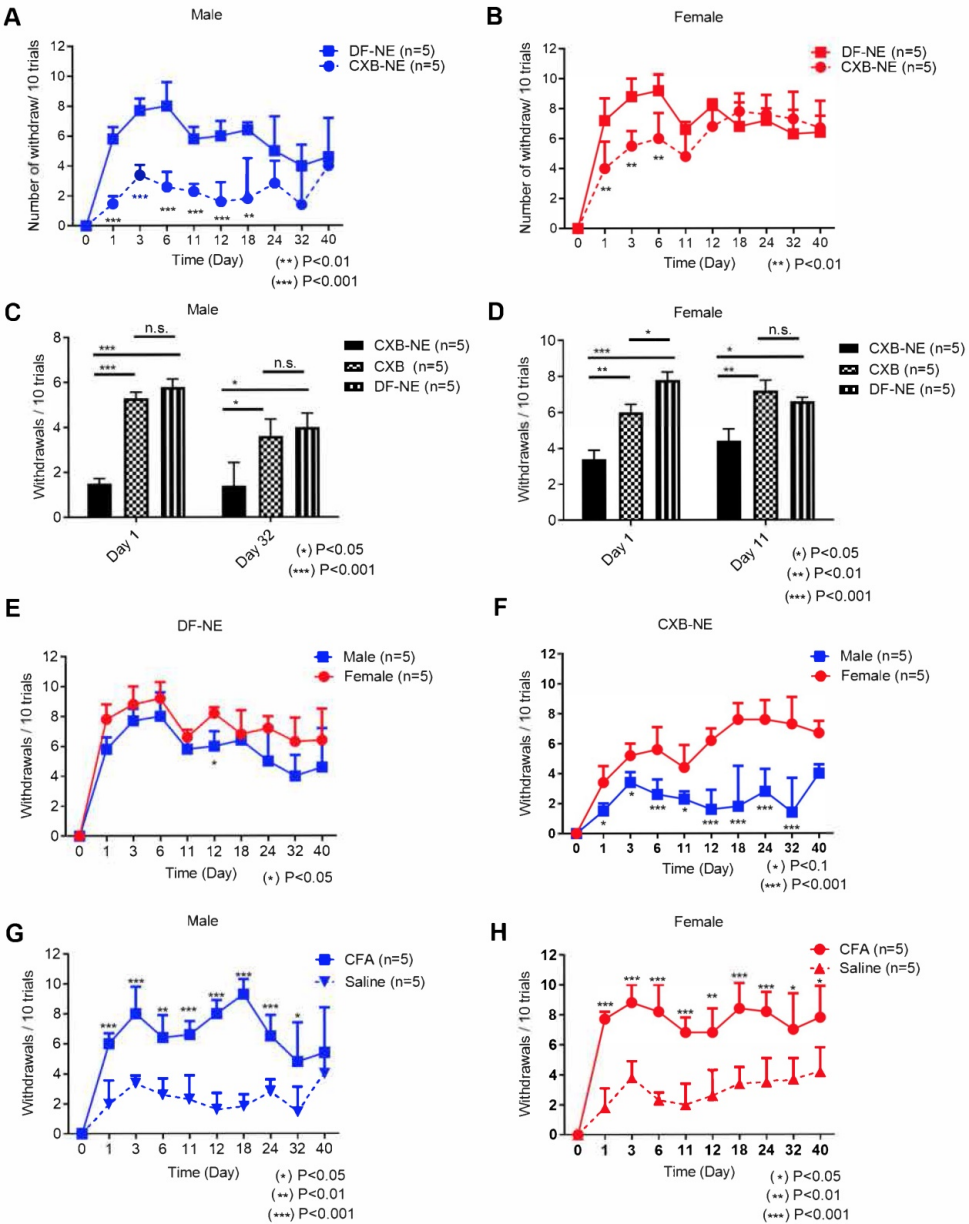
** Mechanical hypersensitivity monitoring for 40 days with von Frey in male and female mice following tail vein injection with 200 µL of CXB-NE or DF-NE 12h prior to i.pl injection with CFA.** A) Mechanical hypersensitivity responses in male mice, show significant difference between mice administered CXB-NE versus DF-NE (control, drug free NE) for 18 days and trend continued up to 32 days post injection; B) Mechanical hypersensitivity responses in female mice show significant difference between mice administered CXB-NE over DF-NE for 6 days; C) CXB-NE show marked improvement in mechanical hypersensitivity in males at days 1 and 32 as compared to free drug (CXB) solution control at matched low dose injected i.v. There is no significant difference between DF-NE and free drug control as both do not produce any pain relief. D) CXB-NE results in statistically significant improvement in pain behavior in females at days 1 and 11 over free drug (CXB) control. E) Male and female mice administered DF-NE reveal no statistical significance in mechanical hypersensitivity during the course of the experiment except at day 12 post CFA injection (*p<0.05). F) Male and female mice reveal significant differences between footpad withdrawal responses. There is a marked difference during the course of the experiment for 32 days showing that CXB-NE results in greater improvement in mechanical hypersensitivity in males than females. G) Mechanical hypersensitivity responses show significant difference for male mice administered CFA i.p. injection vs. saline i.p. injection in right hind footpads. H) Mechanical hypersensitivity responses show significant difference for female mice administered CFA i.p. injection vs. saline i.p. injection in right hind footpads. GraphPad Prism 8 was used for statistical analyses and graphical representation. Data represents average ± SEM, n = 5 across all treatment groups.

Using von Frey assay we found persistent and statistically significant reduction in mechanical hypersensitivity with CXB-NE over DF-NE and drug solution (CXB) controls (Figure 1A-D). In male mice apparent pain control persists for up to 32 days (Figure 1A), while it lasts up to 11 days in females (Figure 1B). Importantly, extended pain behavior change in both males and females is achieved only when CXB is delivered in a NE as CXB-NE and directly to macrophages (Figure 1C-D). Both male and female mice when treated with matched low concentration of CXB delivered as non-targeted, free drug solution showed no improvement in mechanical hypersensitivity for up to 6 days (Figure S3 C-D). This means effectiveness of the low dose of CXB requires targeted delivery to macrophages via NE, which is in agreement with our earlier findings in rat injury models 17.

### Macrophage targeted COX-2 inhibition with CXB-NE demonstrates sex-specific differences

During the course of this study we observed significant differences in males and females in response to CXB-NE treatment over controls (free drug and DF-NE). In male mice, the effects of CXB-NE on improved mechanical hypersensitivity lasted up to 18 days post CFA induction versus DF-NE control (Figure 1A). However, there was an observable trend towards improvement in pain behavior that persisted on days 24 and 32 post CFA induction, yet not statistically significant (Figure 1A). To evaluate the antinociceptive effects more closely we ran two-way ANOVA for each time point (statistical analyses results are provided in the Supplemental information, Tables S1-2). A highly significant (p<0.001) reduction was observed in number of withdrawals when male mice were treated with CXB-NE over free drug (CXB) at days 1 and day 32 (Figure 1C). In female mice, we observed statistically significant differences in mechanical hypersensitivity between CXB-NE treatment group and DF-NE treatment group up to day 6 post CFA induction (Figure 1B). There was an obvious trend between the 2 groups on day 11 and day 12 post CFA induction, yet not statistically significant (Figure 1B). When CXB-NE effects on females were compared to free drug (CXB) treated animals, the mechanical hypersensitivity improvement is sustained until Day 11 (Figure 1D). At early stages of post-CFA induced inflammation, up to day 6, both males and females responded to CXB-NE treatment over free drug and DF-NE as controls demonstrated by significantly reduced mechanical hypersensitivity (Figure S3A-D). Importantly, the CFA induced mechanical hypersensitivity both in males (Figure 1G) and females (Figure 1H) as compared to the saline group. Under these conditions, as shown in Figure S4, no significant difference in withdrawal counts between males and females treated with either CFA alone or saline via i.pl injection was observed. Further, the withdrawal counts remained the same between males and females treated with DF-NE control (Figure 1E). These findings suggest that a robust response was obtained by CFA injection and this response was similar between males and females in this experiment. However, when mechanical hypersensitivity was monitored for males and females treated with drug loaded NE (CXB-NE) over the full course of the 40-day study we found highly significant differences (Figure 1F).

This finding warrants further investigation as it indicates that the sex difference is exclusively tied to targeted delivery of CXB to macrophages and not found in control treatment groups. The sex differences in response to treatment did not seem to be confounded by differences in response to either CFA or saline injection (Figure 1G-H). Based on these findings we concluded that COX-2 inhibition via targeted NE can enable extended pain relief in animal models with marked sex-differences (Figure 1). Further we also showed that targeted delivery is necessary to achieve pain relief with COX-2 inhibitor at a very low dose.

### Whole body NIRF confirms localization of CXB-NE and DF-NE at the site of CFA injection in males and females

**Figure 2:**
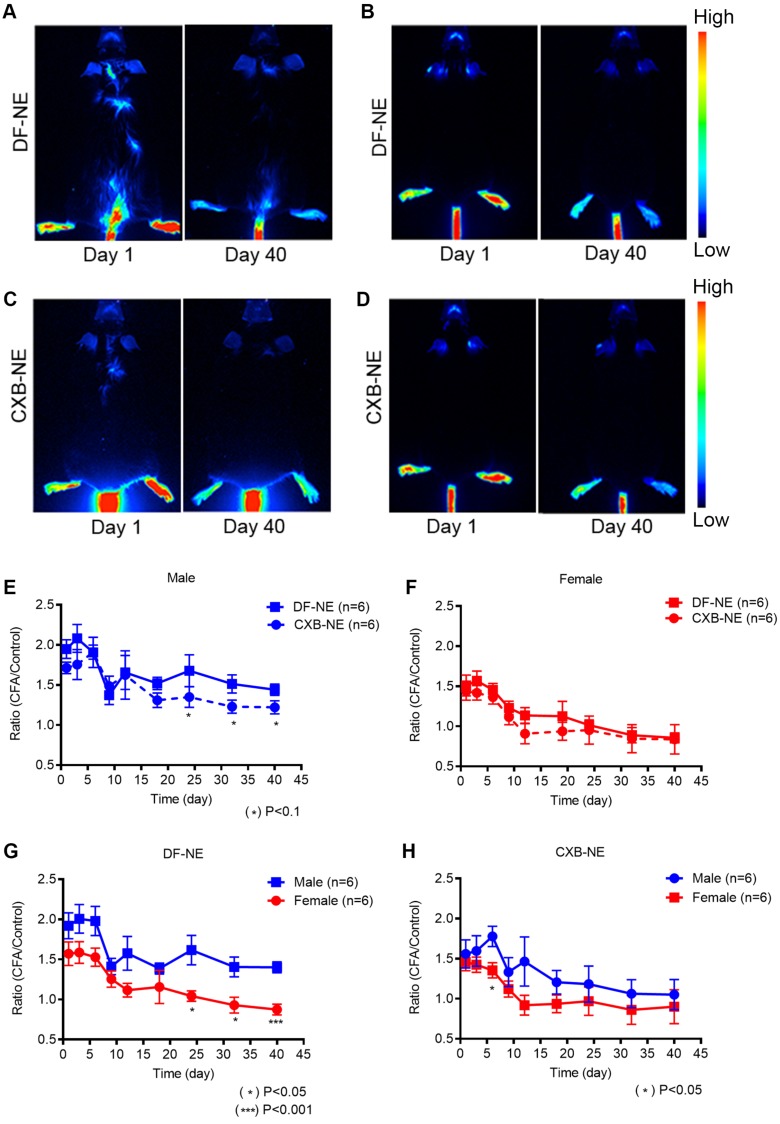
** NIRF whole body imaging of CXB-NE and DF-NE accumulation at the site of inflammation following CFA injection in males and females.** The NEs were injected 12h prior to the CFA induction at the right hind paw in male and female mice. Whole body NIRF images were collected post-CFA induction at distinct time points: days 1, 3, 6, 9, 12, 15, 18, 24, 32, and 40. A-B) Representative NIRF images showing the accumulation of DF-NE (drug free NE) at the site of inflammation (hind right paw) at days 1 and 40 post-CFA induction in males (A) and females (B). C-D) Accumulation of CXB-NE at the site of inflammation (hind right paw) at days 1 and 40 post-CFA injection in males (C) and females (D); E-H) The ratio of quantified fluorescence of inflamed hind right paw footpad is calculated to that of the control (contralateral, left paw) footpad in both between male and females at days 1, 3, 6, 9, 12, 15, 18, 24, 32, and 40 post-CFA. E) DF-NE and CXB-NE associated signal distribution in males over 40 days follow up. CXB-NE treated animals show decreasing signal over time, though not statistically different from DF-NE treatments. This indicate the CXB-NE may impact macrophage infiltration dynamics in males. F) Comparison of signal distribution in DF-NE and CXB-NE treated female mice shows no statistical significance between the treatment groups. G) DF-NE accumulation at the site of CFA insult differs between males and females with a lower overall trend of accumulation in females. There is a lower (statistically significant) signal in females versus males at later time points (Days 24, 32 (p<0.05) and day 40 (p<0.001). H) CXB-NE shows no significant difference in accumulation patterns between males and females over 40 days of follow up. GraphPad Prism 8 was used for statistical analyses and graphical representation. Data represents average ± SEM, n = 5 across all treatment g roups. Two-way ANOVA was used to establish statistical significance between all groups.

High dose of NIRF labeled NEs was used to achieve maximal labeling of macrophages upon i.v. injection produce strong NIRF signal affected region (CFA injection site) as the region of interests (ROI). We found that NIRF fluorescent signal in affected paws, which we hypothesized based on earlier studies that corresponds to macrophage infiltration levels, persists for 40 days post NE injection in both males and females. NIRF whole body images were collected at distinct time points: days 1, 3, 6, 9, 12, 15, 18, 24, 32, and 40 post-CFA induction in males and females injected with DF-NE or CXB-NE. Whole body NIRF images of DF-NE treated male (Figure 2A) and female (Figure 2B) mice and CXB-NE treated males (Figure 2C) and females (Figure 2D) at days 1 and 40, showing earliest and latest time-points. Additional time points collected representative images for all groups are shown in supplemental information (Figure S6). The persistence of NIRF signal, which correlated to the presence of CXB-NE or DF-NE in affected ROIs, at the site of CFA injection, was further confirmed by immunofluorescence of excised inflamed tissues at day 40 (Figure 6, below). To the best of our knowledge, this is the first time NIRF labeled macrophage targeted NEs were used to follow these cells beyond initial acute inflammation and 40 days post CFA insult. Because of the extended follow up duration and single injection used, we want to emphasize that NIRF signal distribution patterns must be interpreted cautiously. We observed no statistically significant differences in NIRF signal in CFA injected hind paws in males (Figure 2E) and females (Figure 2F) between CXB-NE and DF-NE treatment groups. However, we observed interesting differences in distribution of NIRF signal in animals treated with CXB-NE and DF-NE in both males and females (Figure 2E-F). There was a statistically higher signal in male animals treated with DF-NE at later time points (Days 24, 32 and 40), Figure 2G. This sex difference in NIRF signal at CFA treated paw not observed in CXB-NE treated animals across all time points (Days 1-40), Figure 2H.

**Figure 3:**
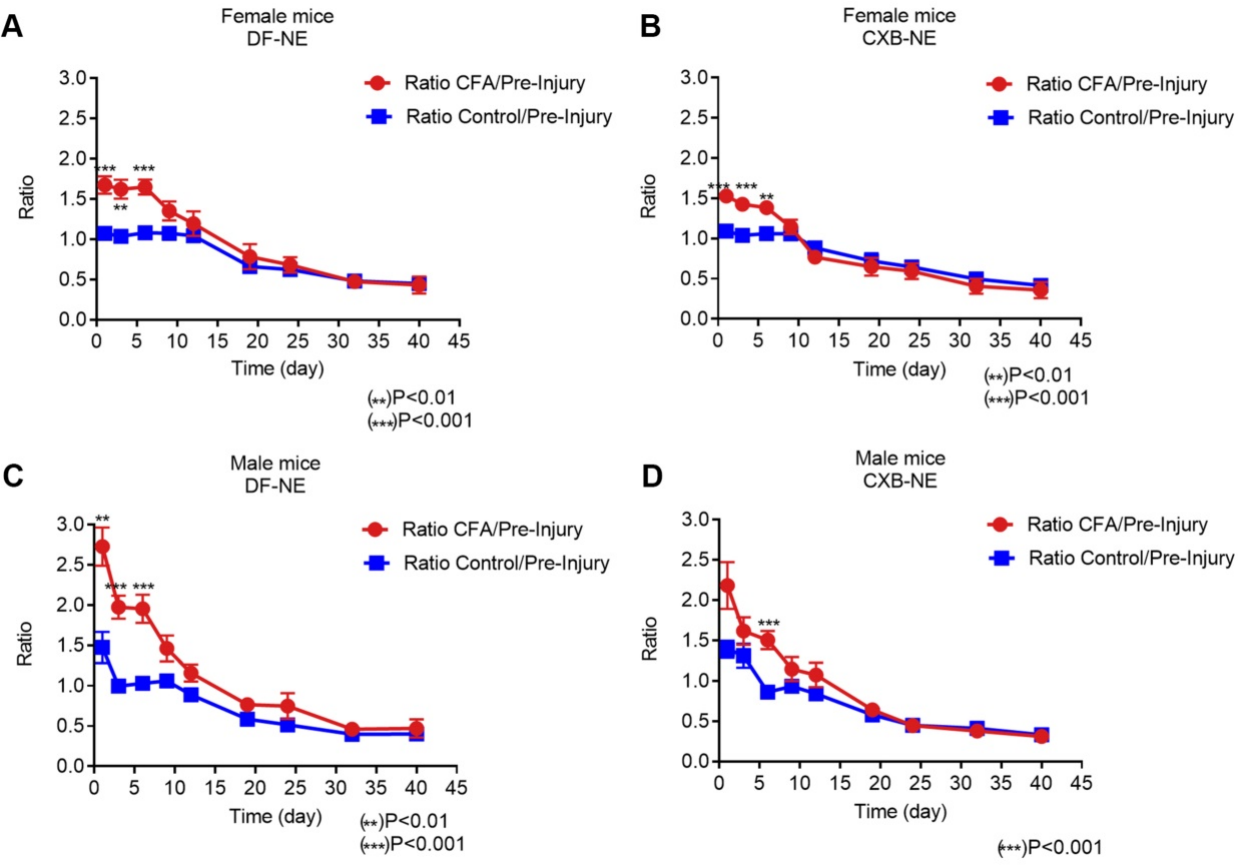
** Whole Body NIRF of CXB-NE and DF-NE at the ratio of CFA footpad/ itself pre-injury in males and females.** A) In DF-NE treated female group, the ratio of CFA/pre-injury showed statistically significant difference at day 1, 3, and 6 post CFA injection compare to the ratio of control/pre-injury; B) In CXB-NE treated female group, the ratio of CFA/pre-injury showed statistically significant difference at day 1, 3, and 6 post CFA injection compare to the ratio of control/pre-injury; C) In DF-NE treated male group, the ratio of CFA/pre-injury showed statistically significant difference at day 1, 3, and 6 post CFA injection compare to the ratio of control/pre-injury; D) In CXB-NE treated male group, the ratio of CFA/pre-injury only showed statistically significant difference at day 6 post CFA injection compare to the ratio of control/pre-injury (Figure 3D).

Furthermore, in order to study the correlation between live NIRF imaging of infiltrating macrophages and CFA-induced inflammation, we plotted WBI data at different time points up to 40 days in both male and female animals administered CXB-NE and DF-NE. To control for inter-subject variability, the fluorescence signal in the injured (CFA) paw at each specific time point was divided by the fluorescence signal in the injured paw prior to CFA injection (CFA/pre-injury). The control (non-injured) footpads were also evaluated using this approach. Female mice treated with either DF-NE or CXB-NE showed statistically significant differences on days 1, 3, and 6 post-CFA injection when comparing the CFA/pre-injury ratio to the control/pre-injury ratio (Figure 3A-B). In the DF-NE treated male group, statistically significant differences were also observed on days 1, 3, and 6 post CFA injection when comparing the CFA/pre-injury ratio to the control/pre-injury ratio (Figure 3C). However, in the CXB-NE treated male group, the ratio of CFA/pre-injury only showed a statistically significant difference on day 6 post CFA injection (Figure 3D). These data demonstrated that CFA injection induced severe inflammation at early time points (days 1, 3, and 6 post injection), which confirms the findings in previous reports 32, 33. Further, the results in Figure 3D suggested that in male mice, treatment with CXB-NE reduces macrophage infiltration in the injured (CFA) footpad.

### Ex vivo NIRF enables quantitative evaluation of tissue distribution of CXB-NE and DF-NE in recipient animals

**Figure 4:**
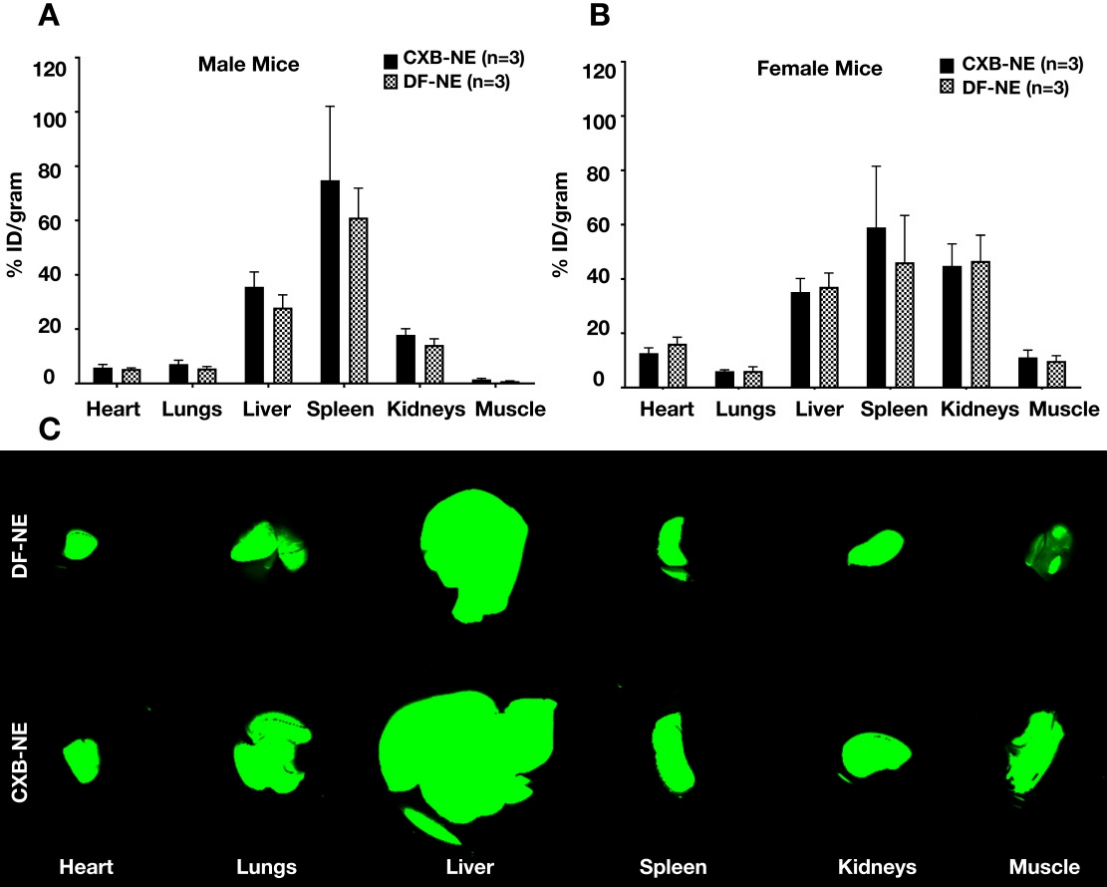
** Biodistribution of theranostic NEs. A.** Representative fluorescence image quantification of indicated organs showing NE biodistribution at 40 days post CFA-induced inflammation in male animals *ex vivo* as evaluated by Odyssey CLx imaging system. **B.** Representative fluorescence image quantification of indicated organs showing NE biodistribution at 40 days post CFA-induced inflammation in female animals *ex vivo* as evaluated by Pearl Trilogy Small Animal Imaging System.** C.** Representative fluorescent images of biodistribution in the organs.

* Ex vivo* NIRF imaging was used to quantify the percent injected dose of NE per gram of organ weight (%ID/g) in male and female mice administered CXB-NE or DF-NE (males, n=6/group; females, n=3/group). We demonstrated that the %ID/g in the heart, lung, liver, spleen, kidneys, and CFA injected leg muscle was not statistically significantly different (p-value<0.05) between the CXB-NE and the DF-NE treatments for both male and female mice (Figure 4A-B). Representative fluorescent images of biodistribution in the studied organs are shown in Figure 4C. The biodistribution study reconfirmed our earlier findings that the liver and spleen accumulate NE the most compared to the other studied non-target organs 19.

These results (Figure 4) were expected because macrophages are present in large quantities in the liver and spleen 34, 35. Though we did not observe a significant difference in the biodistribution between CXB-NE and DF-NE (for both male and female mice), there may still be a biological difference 36. For example, the percentage of inflammatory (M1) macrophages present in the liver and spleen may have decreased in response to treatment with CXB-NE. Previous work has demonstrated that treatment with CXB-NE decreases the percentage of M1 macrophages present at the site of injury 10. This may be the case in off target organs as well.

### Immunohistopathology and Immunofluorescence evaluation at end-point reveals differential inflammatory patterns in hind paws of males and females

**Figure 5:**
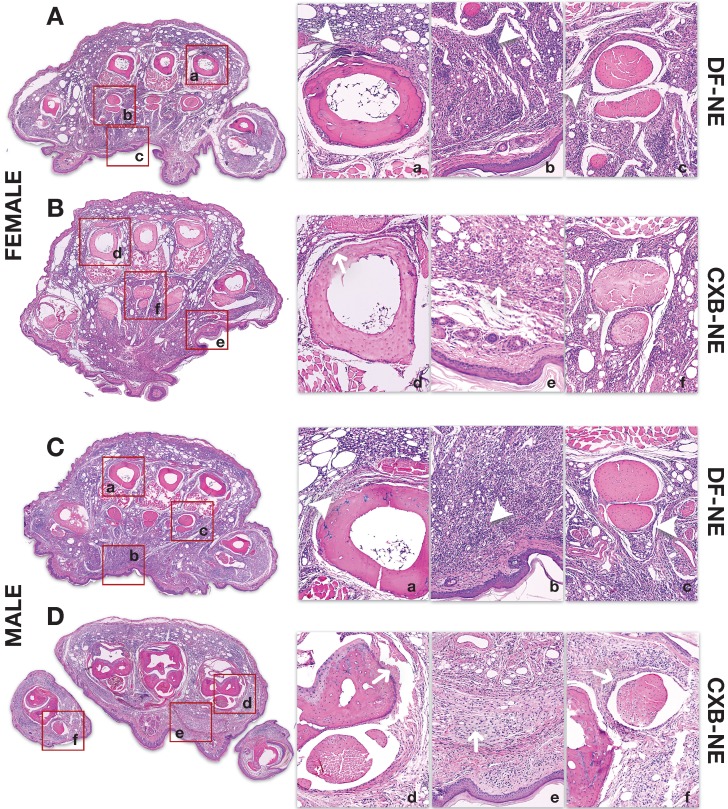
** H&E staining of inflamed paw sections collected at end-point from male and female mice treated with CXB-NE or DF-NE.** A) Coronal Section of Female Mouse Hind Paw at 40 days following CFA Injection: Panel A shows a coronal section of the mid foot in a female mouse receiving DF-NE injection. Significant inflammatory infiltration (*white arrowheads*) is seen in the periosteal zone around the metatarsals (4^th^ metatarsal is shown in [a]), soft tissue (including epidermo-dermal structures, interfaces and adnexa in [b]) and the synovial sheaths of the flexor tendons (the flexor digitorum brevis tendons and sheath are shown for the 2^nd^ metatarsal in [c]) Panel B shows a coronal section of the mid foot in a female mouse receiving CXB-NE injection. There is only partial improvement or resolution of inflammation (*white arrows*) as manifested by infiltration in the periosteal zone around the metatarsals (2^nd^ metatarsal is shown in [d]), soft tissue (including epidermo-dermal structures, interfaces and adnexa in [e]) and the synovial sheaths of the flexor tendons (the flexor digitorum brevis tendons and sheath are shown for the 3^rd^ metatarsal in [f]); B) Coronal Section of Male Mouse Hind Paw at 40 days following CFA Injection: Panel C shows a coronal section of the mid foot in a male mouse receiving DF-NE injection. Significant inflammatory infiltration (*white arrowheads*) is seen in the periosteal zone around the metatarsals (2^nd^ metatarsal is shown in [a]), soft tissue (including epidermo-dermal structures, interfaces and adnexa in [b]) and the synovial sheaths of the flexor tendons (the flexor digitorum brevis tendons and sheath are shown for the 4^th^ metatarsal in [c]) Panel D shows a coronal section of the mid foot in a male mouse receiving CXB-NE injection. Compared to that in the female mouse hind paw, there is marked improvement or resolution of inflammation (*white arrows*) as shown by discernible reduction in infiltration in the periosteal zone around the metatarsals (4^th^ metatarsal is shown in [d]), soft tissue (including epidermo-dermal structures, interfaces and adnexa in [e]) and the synovial sheaths of the flexor tendons (the flexor digitorum brevis tendons and sheath are shown for the 1st metatarsal in [f]).

Male and female mouse hind paws were analyzed by routine histopathology (H&E) and immunofluorescence staining for macrophage specific markers (CD68 and COX-2) at 40 days following CFA injection. We chose coronal sections as they allow superior spatial interpretation and anatomical localization of inflammatory patterns within the hind paw and assessment of variations in degree of immune cell infiltration after CFA injury. Significant inflammation as manifested by diffuse inflammatory cell infiltration is seen in the deep dermis in both female (Figure 5 [DF-NE (Panel A)], and male controls (Figure 5 [DF-NE (Panel C)], In contrast to control subjects, where there was minimal difference in degree of inflammation, at 40 days, there is improvement of inflammation in both sexes, as manifested by resolution of infiltrates (Figure 5 [CXB-NE (Panels B and D)], . Male mice demonstrated more significant resolution of inflammation as compared to female mice with CXB-NE treatment. This effect was particularly prominent in the periosteal and synovial zones as well as in the deep dermis.

**Figure 6:**
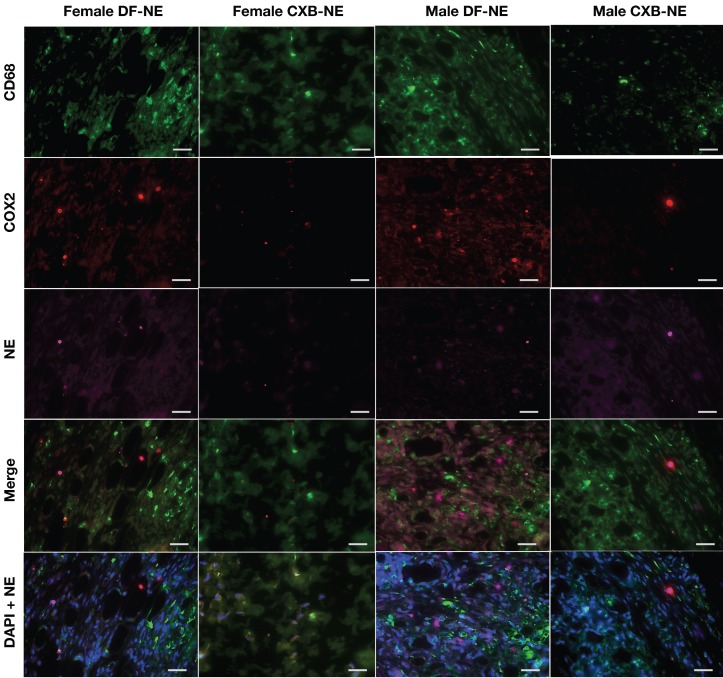
** Representative images of immunofluorescence staining of excised tissues from CFA-treated footpad in males and females on day 40 post CFA induction.** Sections were stained with Rat anti mouse-CD68 (selective macrophage marker, green) and Goat anti mouse-COX-2 (COX-2, red). DF-NE and CXB-NE shown as purple. The merged panel shows the co-localization of CXB-NE or DF-NE with COX-2 expressing macrophages, confirming the persistence of both NEs in affected tissues at day 40 post-CFA injection. The lowermost panel shows DAPI nuclear counterstaining co-localized with NE in both DF-NE and CXB-NE groups, confirming the intracellular (peri-nuclear) localization of NE inside macrophages (CD68 _positive or negative_).

**Figure 7:**
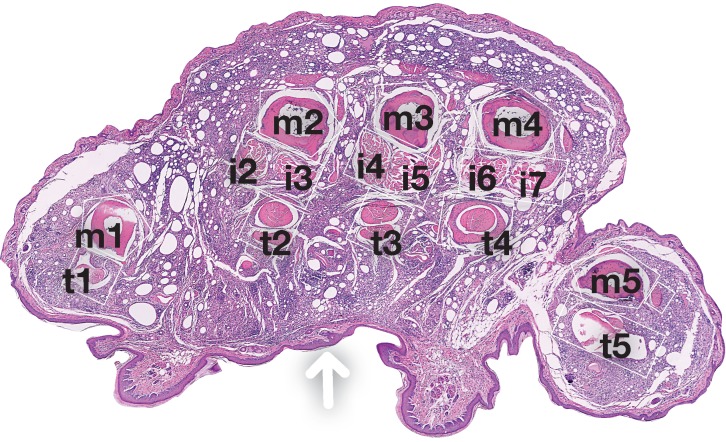
** Coronal section of the mouse hind paw** showing contextual anatomical localization of CFA-induced inflammation. A coronal section of the mid foot is shown with the arrow demonstrating the location of CFA injection on the plantar aspect of the sole of the hind paw. The metatarsals of the big toe (m1), and other toes are labeled (m2, m3, m4, m5). The flexor digitorum brevis (FDB) tendons are shown for each toe (t1 for great toe, and t2, t3, t4, t5 for other toes). The FDB arises from the tendon of plantaris as three slender muscles which pass over into long tendons. Each tendon, at the base of the first phalanx, divides into two and insert on the proximal end of the second phalanx of the second, third and fourth toes. The plantar interossei for the 2nd, 3rd and 4th toe are shown (i2, i3 [2nd digit]; i4, i5 [3rd digit]; i6, i7 [4th digit]). These are small slender muscles on the volar surface of the metatarsals. There are two interossei for 2nd, 3rd and 4th metatarsals, and one each for the great toe and little toe.

Immunofluorescent staining (Figure 6) for CD68 expression showed strong positivity in female control mice as compared to male mice (DF-NE). No significant differences were observed in the levels of COX-2 expression in controls. However, CXB-NE treatment substantially suppressed COX-2 expression in both sexes. The merged panels in both male and female CXB-NE treated mice on Figure 6 demonstrate that there is a persistence of CD68 positive macrophages in the hind paws at 40 days but COX-2 expression is very low or negligible in both sexes. DAPI nuclear counterstaining with NE confirmed the intracellular (peri-nuclear/intracytoplasmic) localization and persistence of NE in macrophages in both DF-NE and CXB-NE groups at 40 days after CFA injection. Figure 7 shows anatomical context to better localize the inflammation which is marked in the periosteal zone of the metatarsals and in the synovial sheaths of the flexor digitorum brevis tendons. There is absent or negligible inflammation in the plantar interossei (interposed between the tendons and the metatarsals.

## Discussion

Monocytes and macrophages are attractive targets for both diagnosis and treatment of inflammation as they are shown to directly contribute to pathology of multiple inflammatory diseases such as osteoarthritis, rheumatoid arthritis and inflammatory bowel disease 30-32. In prior studies we demonstrated that CXB-NEs when delivered intravenously in rat chronic constriction injury (CCI) model, are taken up by macrophages changing their infiltrating patterns as compared to DF-NE 33. We also showed that both CXB-NE and DF-NE co-localize with CD68 positive cells (macrophages) and COX-2 in tissues excised from male mice hind paws treated with CFA at day 4 post-induction 3. Here we extended our imaging studies to 40 days at selected time points after CFA injection in both male and female mice between CXB-NE and DF-NE groups.

Our study confirms that administration of a single dose of CXB-NE significantly reduces CFA-induced mechanical hypersensitivity in both male and female mice for longer than one week (Figure 1A, B). However, this pain reduction was more significant in male mice at later time points (Figure 1D). CXB-NE provided extended improvement in pain behavior in males (32 days, Figure 1A) versus females (11 days, Figure 1B), which may be dependent on targeted delivery of CXB directly to macrophages expressing COX-2 enzyme. This is likely due to extended inhibition of PgE2 release from these cells achieved by prolonged COX-2 exposure to the inhibitor. This effect is achieved by internalized CXB-NE droplets serving as drug depot inside macrophages.

Importantly, CXB administered as a free drug showed no effect in either males or females when administered at matched concentration to that delivered with CXB-NE (Figure 1C-D). This finding strongly suggests that effectiveness of CXB in inflammatory pain can be dramatically improved by incorporation into macrophage targeted NEs. CFA treated paw NIRF signal in female and male mice when administered CXB NE or DFNE, persists up to day 40, Figure 2, while it changes in intensity over time. This finding indicates that targeted delivery of CXB directly to sites of inflammation can be monitored over long period of time using NIRF imaging, where NIRF signal from NE may be used to infer levels of drug accumulation. The value of these findings is to demonstrate the potential for using nanotheranostics, such as the NEs evaluated on this study, as tools for studying macrophage turnover and life-span in chronic inflammation. Further, these data indicate there are macrophage infiltration sex-differences in males and females that warrant further investigation in preclinical models. For example, we found that fluorescent ratio of CFA/control decreases to 1.0 in female mice (Figure 2F) at the same time point (11 days) when hyperalgesia begins to show less of significant effect of CXB-NE treated mice over controls (free drug and/or DF-NE) in female mice (Figure 1B). This was not observed in male mice. The data indicates there are differences in response to COX-2 inhibition in males and females that may correspond to changes in NE labeled macrophage infiltration levels between the sexes.

Immunofluorescence staining (Figure 6) on hind paw samples from Day 40, revealed significantly more CD68 positive macrophages containing phagocytosed NE, in DF-NE treated male mice as compared to females. Tissue counterstaining with DAPI confirmed the intracytoplasmic location of NE in both DF and CXB-NE groups. This finding correlated with a statistically significant increase in NIRF signal intensity in males versus female DF-NE treated mice (Figure 2G). On H&E (Figure 5), we observed increased gross infiltration in inflammatory cells at 40 days after CFA in both male and female mice receiving DF-NE. Despite the visible decrease infiltration at day 40 in both male and female mice receiving CXB-NE, the clearance of infiltrates was much more marked in male mice (Figure 5D [upper and lower panels, d,e,f]).

However, we must acknowledge limitations in approach or analyses in this study that prevented us from making more substantive or empirical assumptions: All mice received only a single injection of NE and a dose-response study was not performed. Thus, it may be hypothesized that newly generated monocytes that differentiated into MDMs and infiltrated to the sites of inflammation late after the DF or CXB-NE injection may not have retained the fluorescent label. Interestingly however, we saw persistence of NE in macrophages even at 40 days in both male and female DF-NE mice (Figure 6, DAPI+NE merge). In contrast, very little NE was present in macrophages in the CXB-NE treated male and female mice at day 40. We are currently investigating if the drug loading affects the pharmacokinetics of the NE. However, the marked resolution of inflammation at day 40 as seen on H&E in both male and female (male > female) mice treated with CXB-NE suggest that the anti-inflammatory effects of CXB may persist long beyond the life-span, turnover and removal of CXB-NE laden macrophages. A significant limitation of this study is that histologic analyses were done only at end point and immunohistochemistry for macrophage specific markers was not performed. Other limitations of this work include (1) different groups of animals were used to perform the pain and the NIRF imaging studies; (2) only one type of pain assay was used. To the best of our knowledge, the presented exploratory, predominantly observational work is the only example of investigating the extended impact (up to 40 days) on pain and inflammation with COX-2 inhibition in MDMs through targeted drug delivery. Further, this is the first, albeit proof-of-concept study where sex differences in macrophage specific COX-2 inhibition were ever investigated with a nanotheranostic approach. We envision future work to include sequential imaging, advanced immunohistopathologic analyses, and additional pain behavior assays beginning at earlier time points and coinciding with crucial time points in the inflammation response to injury. Further studies in large animal models may be necessary to validate biological sex differences in inflammatory pain.

## Conclusion

Our study highlights the potential of nanotheranostics as tools to investigate the complex biology and sex-specific nuances in responses to inflammation and pain. Our results in male and female mice highlight the value of a cell-targeted nanotheranostic approach for in-depth investigation of macrophage behavior in inflammatory diseases. This technology can be used to improve our understanding of the mechanism behind sex differences in macrophage biology, inflammation and response to commonly used drugs such as NSAIDs. A multipronged approach including behavioral testing, *in vivo* real time cell tracking, and* ex vivo* histological analyses such as presented here is critical to improve our understanding of the processes that drive such inflammation. Our work provides key foundational insights into development of better personalized pain management approaches for patients.

## Supplementary Material

Supplementary methods, figures and tables.
