# Supplementary material for: Sex Differences Revealed in a Mouse CFA Inflammation Model with Macrophage Targeted Nanotheranostics
Source: Theranostics. 2020 Jan 1;10(4):1694–707. doi: 10.7150/thno.41309 (PMC6993234; doi:10.7150/thno.41309)
Supplement: Supplementary file 1 — Supplementary methods, figures and tables. [file thnov10p1694s1.pdf]

SUPPLEMENTAL INFORMATION

Sex Differences Revealed in a Mouse CFA Inflammation Model with Macrophage Targeted Nanotheranostics

Lu Liu[1,2], Huseyin Karagoz[3], Michele Herneisey[1,2], Fatih Zor[3], Takaaki Komatsu[4], Shannon Loftus[1,2], Bratislav M. Janjic,[5] Vijay S. Gorantla[3*], Jelena M. Janjic[1,2,*]

[1] Graduate School of Pharmaceutical Sciences, School of Pharmacy, Duquesne University, Pittsburgh, PA, USA

[2] Chronic Pain Research Consortium, Duquesne University, Pittsburgh, PA, US
[4] Department of Surgery, Institute of Regenerative Medicine, Wake Forest University School of Medicine, Winston-Salem, North Carolina, United States

[3] Department of Pharmacology, Daiichi University of Pharmacy, Fukuoka, Japan

[5] NRG Oncology Foundation Inc., Pittsburgh, PA, United States

CO-CORRESPONDING AUTHORS:

*Jelena M. Janjic, PhD
Graduate School of Pharmaceutical Sciences,
Duquesne University
600 Forbes Avenue, 415 Mellon Hall
Pittsburgh, PA 15282, USA
412-396-6369 (Phone)
janjicj@duq.edu (Email)

*Vijay S. Gorantla, MD, PhD, MMM
Department of Surgery
Institute of Regenerative Medicine, Wake Forest University School of Medicine
391 Technology Way
Suite 333 Richard H. Dean Biomedical Building
Winston- Salem, NC 27101, USA
336-713-1494 (Phone)
vgorantl@wakehealth.edu (Email)

Methods

Preparation of nanoemulsions

The nanoemulsions used in this study (celecoxib loaded, CXB-NE and drug-free, DF-NE) were produced following earlier reported protocols in Janjic et al[1, 2]. Briefly, nanoemulsions were prepared by incorporating the near-infrared fluorescent dye, DiR, DiIC18(7) (1,1'-Dioctadecyl-3,3,3',3'-Tetramethylindotricarbocyanine Iodide) (5 mM) with/without celecoxib (2.5 mg/mL) into a hydrocarbon oil. After adding perfluorocarbon and surfactants, the mixture was processed on a microfluidizer (Microfluidics, M110S) following earlier established methods.[1]

Colloidal characterization

Dynamic light scattering (Zetasizer Nano ZS, Malvern Instruments, UK) was used to measure droplet size and zeta potential at 25 °C. Nanoemulsion was dispersed in deionized water at a 1:80 dilution. Size and zeta potential were recorded on the same sample in a cuvette or standard zeta potential cell, respectively. Each sample was analyzed three times with at least 12 runs each.

PgE2 ELISA

RAW 264.7 murine macrophages (ATCC) were plated at 300,000 cells/2mL/well in 6 well plates for 24 hours. Cells were then exposed to the indicated concentrations of nanoemulsion or drug dispersed in full media for 24 hours. The cells were then washed and exposed to serum-free media with or without 500 ng/mL LPS. After 18 hours, the cell culture supernatant was collected and centrifuged at 2000 rpm 4°C for two minutes to remove any cell debris. PgE2 was measured in these samples using the Prostaglandin E2 ELISA Kit – Monoclonal (Cayman Chemical, Ann Arbor USA) according to the manufacturer's instructions.

WBI image acquisition

Pearl Trilogy Small Animal Imaging System, was used to evaluate the whole body live imaging (WBI) of an individual mouse with different treatment and at different time points until the end of the study (40 days post-CFA induction). The animal was anesthetized with 1.5-2.5 % isoflurane/oxygen gas mixture inhalation. Then, the animal was placed on the pre-warmed to 37°C imaging chamber bed at prone position. We anchored the animal's face on the inhale nuzzle where they will continuously receive anesthesia. The animal's hind footpads were placed on each side of the tail, and the palm of hind paws were facing up. This allows the camera to acquire a clean shot of a picture representing the accumulation of nanoemulsions' NIRF signal of the inflamed footpad (hind right) and of the control footpad (hind left).

Results

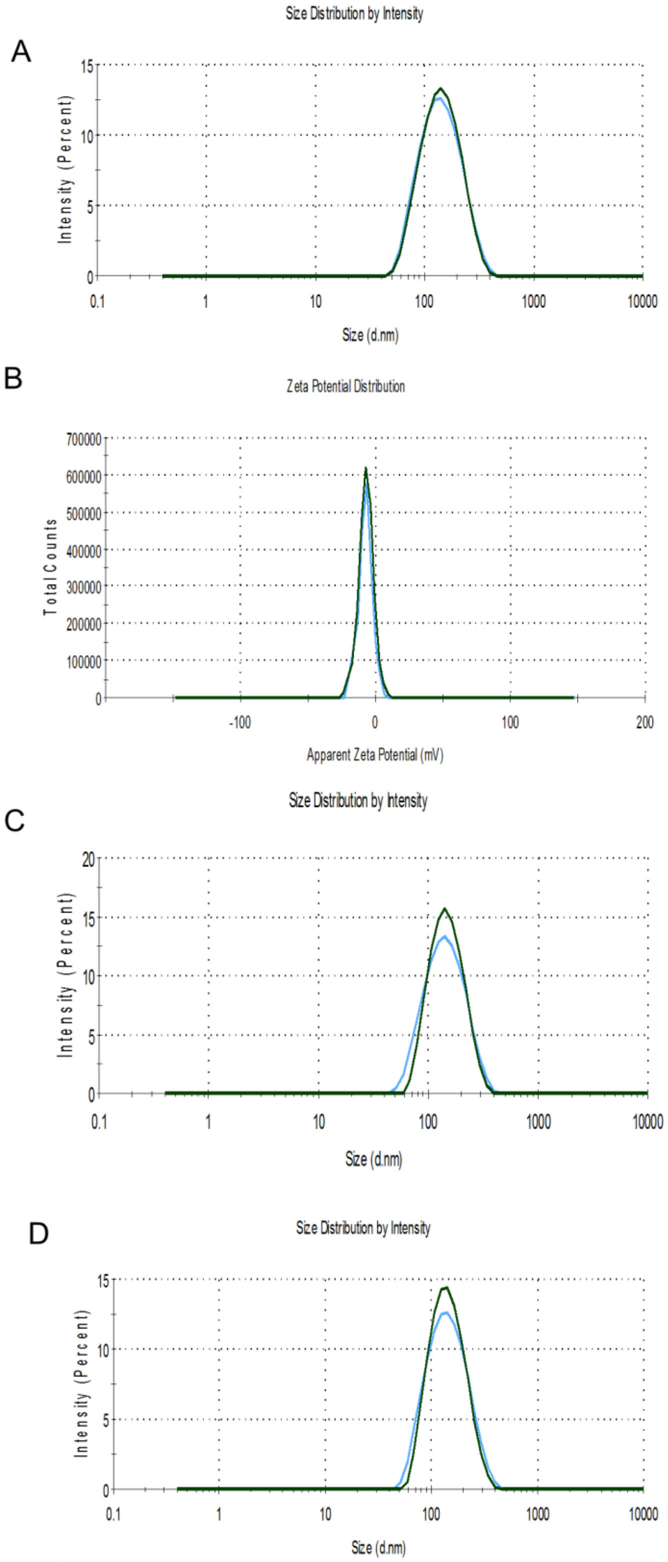

Fig. S1. Comparison of size and zeta potential distributions of the nanoemulsions used in WBI. **A.** Size distribution for CXBNE (green) vs DFNE (blue) 1 day after production and **B.** zeta potential distribution. **C.** Size distribution for CXBNE after 1 day (blue) and 6 months (green) and **D**. Size distribution for DFNE after 1 day (blue) and 6 months (green).

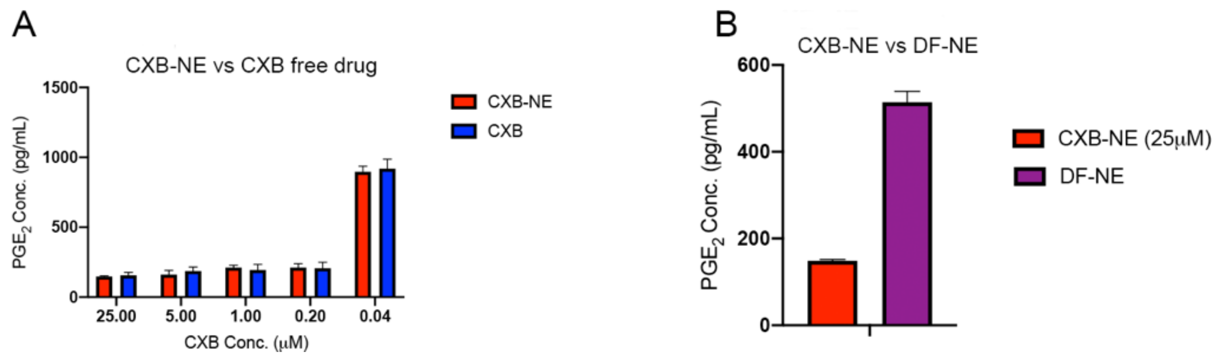

Figure S2. PgE2 ELISA Assay. RAW 264.7 macrophage cells were cultured with increasing concentrations of CXB-NE, DF-NE or free drug (CXB) for 24 h prior LPS exposure for 3h and supernatant collection. **A.**PgE2 production in inhibition from cells treated with a 5-fold dilution dose curve of CXB-NE or DF-NE. **B.** PgE2 production in cells treated with a 5-fold dilution curve of CXB-NE or CXB dissolved in DMSO. Error bars represent standard deviation from triplicate culture aliquots.

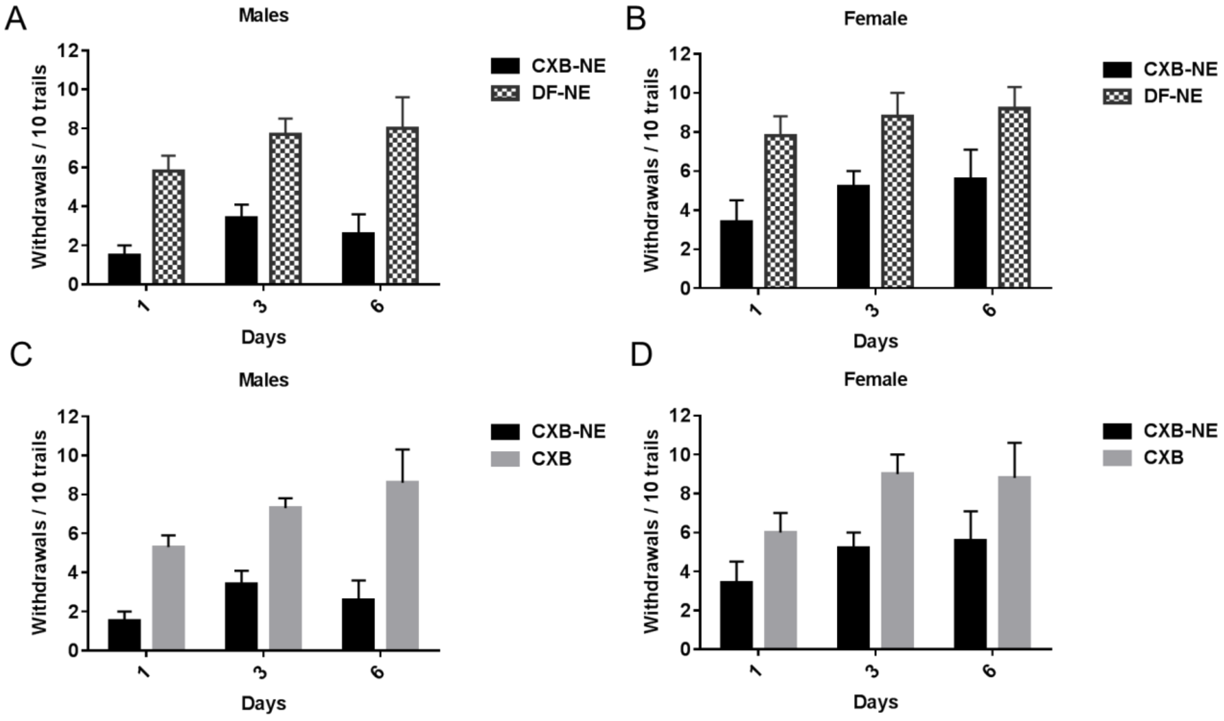

Figure S3. Reanalysis of von Frey results in male and females. First six days post CFA injection mechanical hypersensitivity measurements shows significant pain behavior improvement both in males and females with CXB-NE over controls (CXB or DF-NE).

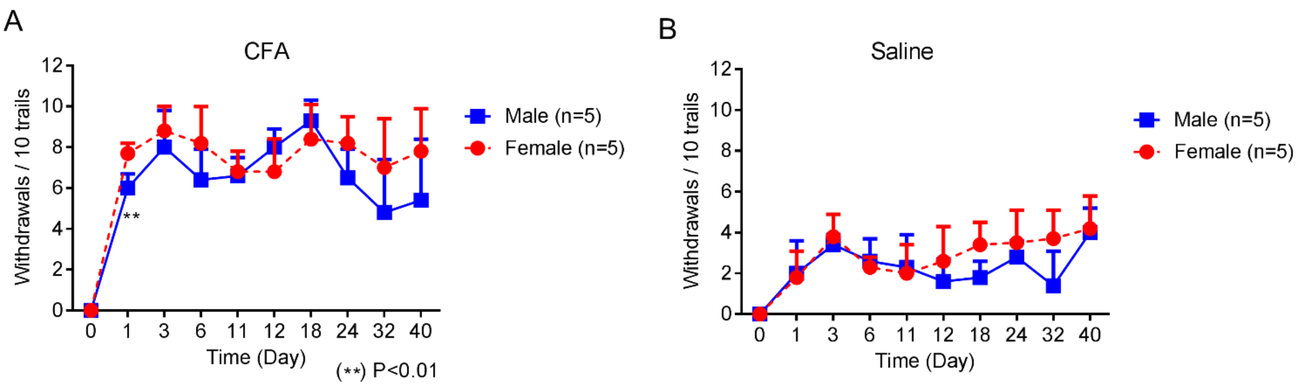

Figure S4. Reanalysis of von Frey results in male and females. A) Mechanical hypersensitivity monitoring for 40 days after CFA i.pl injection in male and female mice. There is no significant difference observed in responses to CFA (B) or saline intraplantar injection between sexes.

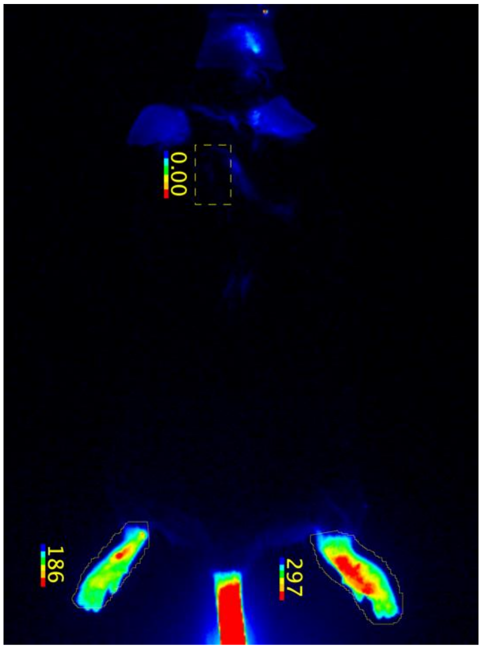

Figure S5. A representation on how we collect the data from whole body imaging (male, DF-NE on day 1 post CFA induction). Images were analyzed and signal quantified using software Image Studio Lite. A rectangular box (area size 960) was placed on the back/shoulder region of each animal across the study, we assigned this selected dark shape as background: signal 0.000. Region of interest (ROIs) were drawn around each CFA-induced footpad and its contralateral footpad as control from toe to heel. Then, we recorded the signals from each footpad as our raw data.

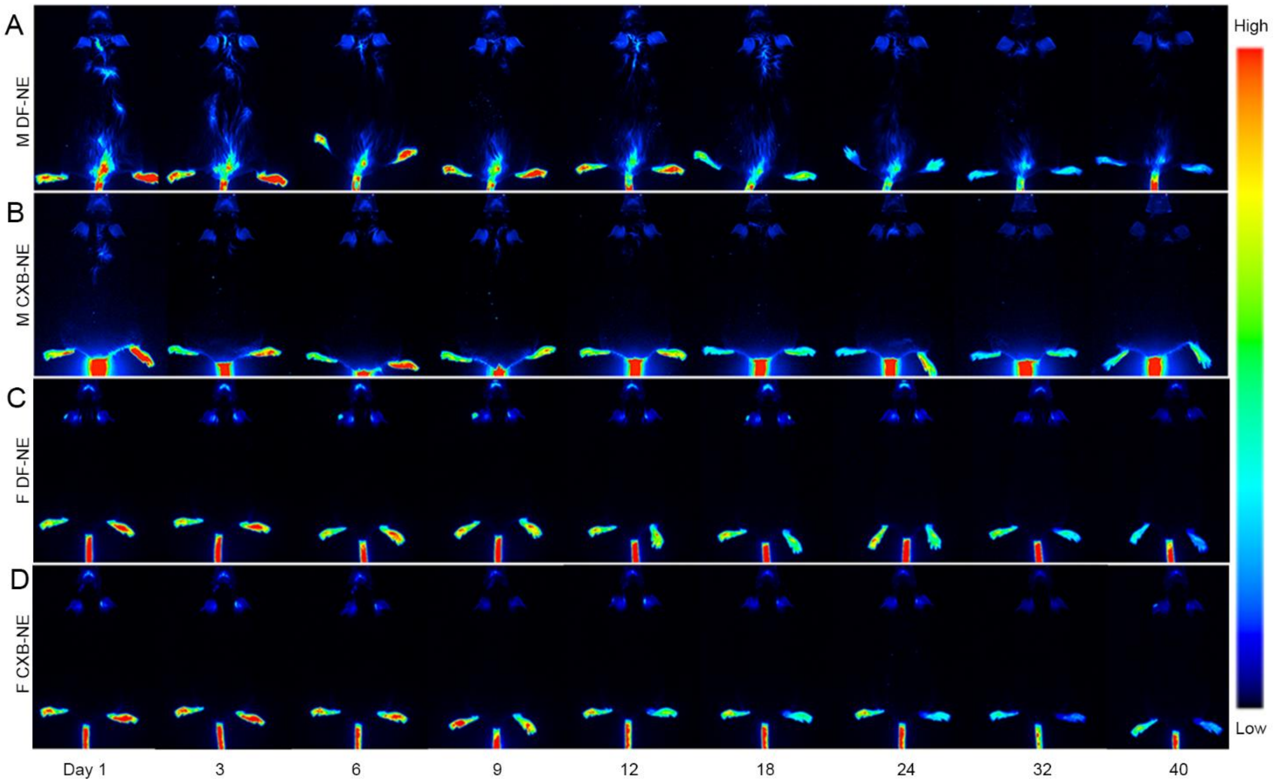

Figure S6. NIRF whole body live images showing the accumulation of nanoemulsion at the site of inflammation (hind right footpad) at different time points (Days 1, 3, 6, 9, 12, 18, 24, 32, and 40 post CFA-induction). The treatment (CXB-NE or DF-NE) were injected *i.v.* via tail vein injection prior to the CFA induction at the right hind paw (50µ intraplantar). A) Representative NIRF images showing the accumulation of DF-NE at the site of inflammation in male mice group from day 1 to day 40 post-CFA induction. B) Representative NIRF images showing the accumulation of CXB-NE at the site of inflammation in male mice group from day 1 to day 40 post-CFA induction. C) Representative NIRF images showing the accumulation of DF-NE at the site of inflammation in female mice group from day 1 to day 40 post-CFA induction. D) Representative NIRF images showing the accumulation of CXB-NE at the site of inflammation in female mice group from day 1 to day 40 post-CFA induction.

References:

1. Janjic, J.M., et al., Low-dose NSAIDs reduce pain via macrophage targeted nanoemulsion delivery to neuroinflammation of the sciatic nerve in rat. J Neuroimmunol, 2018. **318**: p. 72-79.
2. Patel, S.K., et al., Theranostic nanoemulsions for macrophage COX-2 inhibition in a murine inflammation model. Clin Immunol, 2015. **160**(1): p. 59-70.

	A	B	C	D	E	F	G	H	I
1									
2	**Von Frey ASSAY in MALE MICE**								
3									
4	Table Analyzed	Males - vonFrey							
5									
6	Two-way ANOVA	Ordinary							
7	Alpha	0.05							
8									
9	Source of Variation	% of total variati	P value	P value summar	Significant?				
10	Interaction	12.97	< 0.0001	****	Yes				
11	Row Factor	31.84	< 0.0001	****	Yes				
12	Column Factor	35.89	< 0.0001	****	Yes				
13									
14	ANOVA table	SS	DF	MS	F (DFn, DFd)	P value			
15	Interaction	267.3	36	7.426	F (36, 200) = 3.	P < 0.0001			
16	Row Factor	656.4	9	72.93	F (9, 200) = 36.	P < 0.0001			
17	Column Factor	739.9	4	185	F (4, 200) = 92.	P < 0.0001			
18	Residual	398.1	200	1.99					
19									
20									
21	Within each row, compare columns (simple effects within rows)								
22									
23	Number of families	10					Number of missing values	0	
24	Number of comparisons per family	10							
25	Alpha	0.05							
26									
27	**Tukey's multiple comparisons test**	Mean Diff.	95% CI of diff.	Significant?	Summary				
28	**1**								
29	Saline vs. CXB-NE (CFA)	0.5	-1.956 to 2.956	No	ns				
30	Saline vs. CFA	-4	-6.456 to -1.544	Yes	***				
31	Saline vs. CXB (CFA)	-3.3	-5.756 to -0.8437	Yes	**				
32	Saline vs. DF-NE (CFA)	-3.8	-6.256 to -1.344	Yes	***				
33	CXB-NE (CFA) vs. CFA	-4.5	-6.956 to -2.044	Yes	****				
34	**CXB-NE (CFA) vs. CXB (CFA)**	**-3.8**	**-6.256 to -1.344**	**Yes**	***				
35	**CXB-NE (CFA) vs. DF-NE (CFA)**	**-4.3**	**-6.756 to -1.844**	**Yes**	****				
36	CFA vs. CXB (CFA)	0.7	-1.756 to 3.156	No	ns	free drug innefective - no change vs untreated (CFA alone)			
37	CFA vs. DF-NE (CFA)	0.2	-2.256 to 2.656	No	ns	drug-free nanoemulsion ineffective			
38	CXB (CFA) vs. DF-NE (CFA)	-0.5	-2.956 to 1.956	No	ns				
39									
40	**3**								
41	Saline vs. CXB-NE (CFA)	0	-2.456 to 2.456	No	ns				
42	Saline vs. CFA	-4.6	-7.056 to -2.144	Yes	****				
43	Saline vs. CXB (CFA)	-3.9	-6.356 to -1.444	Yes	***				
44	Saline vs. DF-NE (CFA)	-4.3	-6.756 to -1.844	Yes	****				
45	**CXB-NE (CFA) vs. CFA**	**-4.6**	**-7.056 to -2.144**	**Yes**	****				
46	**CXB-NE (CFA) vs. CXB (CFA)**	**-3.9**	**-6.356 to -1.444**	**Yes**	***				
47	**CXB-NE (CFA) vs. DF-NE (CFA)**	**-4.3**	**-6.756 to -1.844**	**Yes**	****				
48	CFA vs. CXB (CFA)	0.7	-1.756 to 3.156	No	ns				
49	CFA vs. DF-NE (CFA)	0.3	-2.156 to 2.756	No	ns				
50	CXB (CFA) vs. DF-NE (CFA)	-0.4	-2.856 to 2.056	No	ns				
51									
52	**6**								
53	Saline vs. CXB-NE (CFA)	0	-2.456 to 2.456	No	ns				
54	Saline vs. CFA	-3.8	-6.256 to -1.344	Yes	***				
55	Saline vs. CXB (CFA)	-6	-8.456 to -3.544	Yes	****				
56	Saline vs. DF-NE (CFA)	-5.4	-7.856 to -2.944	Yes	****				
57	**CXB-NE (CFA) vs. CFA**	**-3.8**	**-6.256 to -1.344**	**Yes**	***				
58	**CXB-NE (CFA) vs. CXB (CFA)**	**-6**	**-8.456 to -3.544**	**Yes**	****				
59	**CXB-NE (CFA) vs. DF-NE (CFA)**	**-5.4**	**-7.856 to -2.944**	**Yes**	****				
60	CFA vs. CXB (CFA)	-2.2	-4.656 to 0.2563	No	ns				
61	CFA vs. DF-NE (CFA)	-1.6	-4.056 to 0.8563	No	ns				
62	CXB (CFA) vs. DF-NE (CFA)	0.6	-1.856 to 3.056	No	ns				
63									
64	**11**								
65	Saline vs. CXB-NE (CFA)	0	-2.456 to 2.456	No	ns				
66	Saline vs. CFA	-4.3	-6.756 to -1.844	Yes	****				
67	Saline vs. CXB (CFA)	-3.5	-5.956 to -1.044	Yes	**				
68	Saline vs. DF-NE (CFA)	-3.5	-5.956 to -1.044	Yes	**				
69	**CXB-NE (CFA) vs. CFA**	**-4.3**	**-6.756 to -1.844**	**Yes**	****				
70	**CXB-NE (CFA) vs. CXB (CFA)**	**-3.5**	**-5.956 to -1.044**	**Yes**	**				
71	**CXB-NE (CFA) vs. DF-NE (CFA)**	**-3.5**	**-5.956 to -1.044**	**Yes**	**				
72	CFA vs. CXB (CFA)	0.8	-1.656 to 3.256	No	ns				
73	CFA vs. DF-NE (CFA)	0.8	-1.656 to 3.256	No	ns				
74	CXB (CFA) vs. DF-NE (CFA)	0	-2.456 to 2.456	No	ns				
75									
76	**12**								
77	Saline vs. CXB-NE (CFA)	0	-2.456 to 2.456	No	ns				
78	Saline vs. CFA	-6.4	-8.856 to -3.944	Yes	****				
79	Saline vs. CXB (CFA)	-6.6	-9.056 to -4.144	Yes	****				
80	Saline vs. DF-NE (CFA)	-4.4	-6.856 to -1.944	Yes	****				
81	**CXB-NE (CFA) vs. CFA**	**-6.4**	**-8.856 to -3.944**	**Yes**	****				
82	**CXB-NE (CFA) vs. CXB (CFA)**	**-6.6**	**-9.056 to -4.144**	**Yes**	****				

	A	B	C	D	E	F	G	H	I
83	**CXB-NE (CFA) vs. DF-NE (CFA)**	**-4.4**	**-6.856 to -1.944**	**Yes**	********				
84	CFA vs. CXB (CFA)	-0.2	-2.656 to 2.256	No	ns				
85	CFA vs. DF-NE (CFA)	2	-0.4563 to 4.456	No	ns				
86	CXB (CFA) vs. DF-NE (CFA)	2.2	-0.2563 to 4.656	No	ns				
87									
88	18								
89	Saline vs. CXB-NE (CFA)	0	-2.456 to 2.456	No	ns				
90	Saline vs. CFA	-7.5	-9.956 to -5.044	Yes	****				
91	Saline vs. CXB (CFA)	-5.2	-7.656 to -2.744	Yes	****				
92	Saline vs. DF-NE (CFA)	-4.6	-7.056 to -2.144	Yes	****				
93	**CXB-NE (CFA) vs. CFA**	**-7.5**	**-9.956 to -5.044**	**Yes**	********				
94	**CXB-NE (CFA) vs. CXB (CFA)**	**-5.2**	**-7.656 to -2.744**	**Yes**	********				
95	**CXB-NE (CFA) vs. DF-NE (CFA)**	**-4.6**	**-7.056 to -2.144**	**Yes**	********				
96	CFA vs. CXB (CFA)	2.3	-0.1563 to 4.756	No	ns				
97	CFA vs. DF-NE (CFA)	2.9	0.4437 to 5.356	Yes	*				
98	CXB (CFA) vs. DF-NE (CFA)	0.6	-1.856 to 3.056	No	ns				
99									
100	24								
101	Saline vs. CXB-NE (CFA)	0	-2.456 to 2.456	No	ns				
102	Saline vs. CFA	-3.7	-6.156 to -1.244	Yes	***				
103	Saline vs. CXB (CFA)	-2.2	-4.656 to 0.2563	No	ns				
104	Saline vs. DF-NE (CFA)	-2.2	-4.656 to 0.2563	No	ns				
105	**CXB-NE (CFA) vs. CFA**	**-3.7**	**-6.156 to -1.244**	**Yes**	*******				
106	**CXB-NE (CFA) vs. CXB (CFA)**	**-2.2**	**-4.656 to 0.2563**	**No**	**ns**				
107	**CXB-NE (CFA) vs. DF-NE (CFA)**	**-2.2**	**-4.656 to 0.2563**	**No**	**ns**				
108	CFA vs. CXB (CFA)	1.5	-0.9563 to 3.956	No	ns				
109	CFA vs. DF-NE (CFA)	1.5	-0.9563 to 3.956	No	ns				
110	CXB (CFA) vs. DF-NE (CFA)	0	-2.456 to 2.456	No	ns				
111									
112	32								
113	Saline vs. CXB-NE (CFA)	0	-2.456 to 2.456	No	ns				
114	Saline vs. CFA	-3.4	-5.856 to -0.9437	Yes	**				
115	Saline vs. CXB (CFA)	-2.2	-4.656 to 0.2563	No	ns				
116	Saline vs. DF-NE (CFA)	-2.6	-5.056 to -0.1437	Yes	*				
117	**CXB-NE (CFA) vs. CFA**	**-3.4**	**-5.856 to -0.9437**	**Yes**	******				
118	**CXB-NE (CFA) vs. CXB (CFA)**	**-2.2**	**-4.656 to 0.2563**	**No**	**ns**				
119	**CXB-NE (CFA) vs. DF-NE (CFA)**	**-2.6**	**-5.056 to -0.1437**	**Yes**	*****				
120	CFA vs. CXB (CFA)	1.2	-1.256 to 3.656	No	ns				
121	CFA vs. DF-NE (CFA)	0.8	-1.656 to 3.256	No	ns				
122	CXB (CFA) vs. DF-NE (CFA)	-0.4	-2.856 to 2.056	No	ns				
123									
124	40								
125	Saline vs. CXB-NE (CFA)	0	-2.456 to 2.456	No	ns				
126	Saline vs. CFA	-1.4	-3.856 to 1.056	No	ns				
127	Saline vs. CXB (CFA)	0	-2.456 to 2.456	No	ns				
128	Saline vs. DF-NE (CFA)	-0.6	-3.056 to 1.856	No	ns				
129	CXB-NE (CFA) vs. CFA	-1.4	-3.856 to 1.056	No	ns				
130	CXB-NE (CFA) vs. CXB (CFA)	0	-2.456 to 2.456	No	ns				
131	CXB-NE (CFA) vs. DF-NE (CFA)	-0.6	-3.056 to 1.856	No	ns				
132	CFA vs. CXB (CFA)	1.4	-1.056 to 3.856	No	ns				
133	CFA vs. DF-NE (CFA)	0.8	-1.656 to 3.256	No	ns				
134	CXB (CFA) vs. DF-NE (CFA)	-0.6	-3.056 to 1.856	No	ns				
135									
136									
137	Test details	Mean 1	Mean 2	Mean Diff.	SE of diff.	N1	N2	q	DF
138									
139	0								
140	Saline vs. CXB-NE (CFA)	0	0	0	0.8923	5	5	0	200
141	Saline vs. CFA	0	0	0	0.8923	5	5	0	200
142	Saline vs. CXB (CFA)	0	0	0	0.8923	5	5	0	200
143	Saline vs. DF-NE (CFA)	0	0	0	0.8923	5	5	0	200
144	CXB-NE (CFA) vs. CFA	0	0	0	0.8923	5	5	0	200
145	CXB-NE (CFA) vs. CXB (CFA)	0	0	0	0.8923	5	5	0	200
146	CXB-NE (CFA) vs. DF-NE (CFA)	0	0	0	0.8923	5	5	0	200
147	CFA vs. CXB (CFA)	0	0	0	0.8923	5	5	0	200
148	CFA vs. DF-NE (CFA)	0	0	0	0.8923	5	5	0	200
149	CXB (CFA) vs. DF-NE (CFA)	0	0	0	0.8923	5	5	0	200
150									
151	1								
152	Saline vs. CXB-NE (CFA)	2	1.5	0.5	0.8923	5	5	0.7925	200
153	Saline vs. CFA	2	6	-4	0.8923	5	5	6.34	200
154	Saline vs. CXB (CFA)	2	5.3	-3.3	0.8923	5	5	5.23	200
155	Saline vs. DF-NE (CFA)	2	5.8	-3.8	0.8923	5	5	6.023	200
156	CXB-NE (CFA) vs. CFA	1.5	6	-4.5	0.8923	5	5	7.132	200
157	CXB-NE (CFA) vs. CXB (CFA)	1.5	5.3	-3.8	0.8923	5	5	6.023	200
158	CXB-NE (CFA) vs. DF-NE (CFA)	1.5	5.8	-4.3	0.8923	5	5	6.815	200
159	CFA vs. CXB (CFA)	6	5.3	0.7	0.8923	5	5	1.109	200
160	CFA vs. DF-NE (CFA)	6	5.8	0.2	0.8923	5	5	0.317	200
161	CXB (CFA) vs. DF-NE (CFA)	5.3	5.8	-0.5	0.8923	5	5	0.7925	200
162									
163	3								
164	Saline vs. CXB-NE (CFA)	3.4	3.4	0	0.8923	5	5	0	200

	A	B	C	D	E	F	G	H	I
165	Saline vs. CFA	3.4	8	-4.6	0.8923	5	5	7.291	200
166	Saline vs. CXB (CFA)	3.4	7.3	-3.9	0.8923	5	5	6.181	200
167	Saline vs. DF-NE (CFA)	3.4	7.7	-4.3	0.8923	5	5	6.815	200
168	CXB-NE (CFA) vs. CFA	3.4	8	-4.6	0.8923	5	5	7.291	200
169	CXB-NE (CFA) vs. CXB (CFA)	3.4	7.3	-3.9	0.8923	5	5	6.181	200
170	CXB-NE (CFA) vs. DF-NE (CFA)	3.4	7.7	-4.3	0.8923	5	5	6.815	200
171	CFA vs. CXB (CFA)	8	7.3	0.7	0.8923	5	5	1.109	200
172	CFA vs. DF-NE (CFA)	8	7.7	0.3	0.8923	5	5	0.4755	200
173	CXB (CFA) vs. DF-NE (CFA)	7.3	7.7	-0.4	0.8923	5	5	0.634	200
174									
175	6								
176	Saline vs. CXB-NE (CFA)	2.6	2.6	0	0.8923	5	5	0	200
177	Saline vs. CFA	2.6	6.4	-3.8	0.8923	5	5	6.023	200
178	Saline vs. CXB (CFA)	2.6	8.6	-6	0.8923	5	5	9.51	200
179	Saline vs. DF-NE (CFA)	2.6	8	-5.4	0.8923	5	5	8.559	200
180	CXB-NE (CFA) vs. CFA	2.6	6.4	-3.8	0.8923	5	5	6.023	200
181	CXB-NE (CFA) vs. CXB (CFA)	2.6	8.6	-6	0.8923	5	5	9.51	200
182	CXB-NE (CFA) vs. DF-NE (CFA)	2.6	8	-5.4	0.8923	5	5	8.559	200
183	CFA vs. CXB (CFA)	6.4	8.6	-2.2	0.8923	5	5	3.487	200
184	CFA vs. DF-NE (CFA)	6.4	8	-1.6	0.8923	5	5	2.536	200
185	CXB (CFA) vs. DF-NE (CFA)	8.6	8	0.6	0.8923	5	5	0.951	200
186									
187	11								
188	Saline vs. CXB-NE (CFA)	2.3	2.3	0	0.8923	5	5	0	200
189	Saline vs. CFA	2.3	6.6	-4.3	0.8923	5	5	6.815	200
190	Saline vs. CXB (CFA)	2.3	5.8	-3.5	0.8923	5	5	5.547	200
191	Saline vs. DF-NE (CFA)	2.3	5.8	-3.5	0.8923	5	5	5.547	200
192	CXB-NE (CFA) vs. CFA	2.3	6.6	-4.3	0.8923	5	5	6.815	200
193	CXB-NE (CFA) vs. CXB (CFA)	2.3	5.8	-3.5	0.8923	5	5	5.547	200
194	CXB-NE (CFA) vs. DF-NE (CFA)	2.3	5.8	-3.5	0.8923	5	5	5.547	200
195	CFA vs. CXB (CFA)	6.6	5.8	0.8	0.8923	5	5	1.268	200
196	CFA vs. DF-NE (CFA)	6.6	5.8	0.8	0.8923	5	5	1.268	200
197	CXB (CFA) vs. DF-NE (CFA)	5.8	5.8	0	0.8923	5	5	0	200
198									
199	12								
200	Saline vs. CXB-NE (CFA)	1.6	1.6	0	0.8923	5	5	0	200
201	Saline vs. CFA	1.6	8	-6.4	0.8923	5	5	10.14	200
202	Saline vs. CXB (CFA)	1.6	8.2	-6.6	0.8923	5	5	10.46	200
203	Saline vs. DF-NE (CFA)	1.6	6	-4.4	0.8923	5	5	6.974	200
204	CXB-NE (CFA) vs. CFA	1.6	8	-6.4	0.8923	5	5	10.14	200
205	CXB-NE (CFA) vs. CXB (CFA)	1.6	8.2	-6.6	0.8923	5	5	10.46	200
206	CXB-NE (CFA) vs. DF-NE (CFA)	1.6	6	-4.4	0.8923	5	5	6.974	200
207	CFA vs. CXB (CFA)	8	8.2	-0.2	0.8923	5	5	0.317	200
208	CFA vs. DF-NE (CFA)	8	6	2	0.8923	5	5	3.17	200
209	CXB (CFA) vs. DF-NE (CFA)	8.2	6	2.2	0.8923	5	5	3.487	200
210									
211	18								
212	Saline vs. CXB-NE (CFA)	1.8	1.8	0	0.8923	5	5	0	200
213	Saline vs. CFA	1.8	9.3	-7.5	0.8923	5	5	11.89	200
214	Saline vs. CXB (CFA)	1.8	7	-5.2	0.8923	5	5	8.242	200
215	Saline vs. DF-NE (CFA)	1.8	6.4	-4.6	0.8923	5	5	7.291	200
216	CXB-NE (CFA) vs. CFA	1.8	9.3	-7.5	0.8923	5	5	11.89	200
217	CXB-NE (CFA) vs. CXB (CFA)	1.8	7	-5.2	0.8923	5	5	8.242	200
218	CXB-NE (CFA) vs. DF-NE (CFA)	1.8	6.4	-4.6	0.8923	5	5	7.291	200
219	CFA vs. CXB (CFA)	9.3	7	2.3	0.8923	5	5	3.645	200
220	CFA vs. DF-NE (CFA)	9.3	6.4	2.9	0.8923	5	5	4.596	200
221	CXB (CFA) vs. DF-NE (CFA)	7	6.4	0.6	0.8923	5	5	0.951	200
222									
223	24								
224	Saline vs. CXB-NE (CFA)	2.8	2.8	0	0.8923	5	5	0	200
225	Saline vs. CFA	2.8	6.5	-3.7	0.8923	5	5	5.864	200
226	Saline vs. CXB (CFA)	2.8	5	-2.2	0.8923	5	5	3.487	200
227	Saline vs. DF-NE (CFA)	2.8	5	-2.2	0.8923	5	5	3.487	200
228	CXB-NE (CFA) vs. CFA	2.8	6.5	-3.7	0.8923	5	5	5.864	200
229	CXB-NE (CFA) vs. CXB (CFA)	2.8	5	-2.2	0.8923	5	5	3.487	200
230	CXB-NE (CFA) vs. DF-NE (CFA)	2.8	5	-2.2	0.8923	5	5	3.487	200
231	CFA vs. CXB (CFA)	6.5	5	1.5	0.8923	5	5	2.377	200
232	CFA vs. DF-NE (CFA)	6.5	5	1.5	0.8923	5	5	2.377	200
233	CXB (CFA) vs. DF-NE (CFA)	5	5	0	0.8923	5	5	0	200
234									
235	32								
236	Saline vs. CXB-NE (CFA)	1.4	1.4	0	0.8923	5	5	0	200
237	Saline vs. CFA	1.4	4.8	-3.4	0.8923	5	5	5.389	200
238	Saline vs. CXB (CFA)	1.4	3.6	-2.2	0.8923	5	5	3.487	200
239	Saline vs. DF-NE (CFA)	1.4	4	-2.6	0.8923	5	5	4.121	200
240	CXB-NE (CFA) vs. CFA	1.4	4.8	-3.4	0.8923	5	5	5.389	200
241	CXB-NE (CFA) vs. CXB (CFA)	1.4	3.6	-2.2	0.8923	5	5	3.487	200
242	CXB-NE (CFA) vs. DF-NE (CFA)	1.4	4	-2.6	0.8923	5	5	4.121	200
243	CFA vs. CXB (CFA)	4.8	3.6	1.2	0.8923	5	5	1.902	200
244	CFA vs. DF-NE (CFA)	4.8	4	0.8	0.8923	5	5	1.268	200
245	CXB (CFA) vs. DF-NE (CFA)	3.6	4	-0.4	0.8923	5	5	0.634	200
246									

	A	B	C	D	E	F	G	H	I
247	40								
248	Saline vs. CXB-NE (CFA)	4	4	0	0.8923	5	5	0	200
249	Saline vs. CFA	4	5.4	-1.4	0.8923	5	5	2.219	200
250	Saline vs. CXB (CFA)	4	4	0	0.8923	5	5	0	200
251	Saline vs. DF-NE (CFA)	4	4.6	-0.6	0.8923	5	5	0.951	200
252	CXB-NE (CFA) vs. CFA	4	5.4	-1.4	0.8923	5	5	2.219	200
253	CXB-NE (CFA) vs. CXB (CFA)	4	4	0	0.8923	5	5	0	200
254	CXB-NE (CFA) vs. DF-NE (CFA)	4	4.6	-0.6	0.8923	5	5	0.951	200
255	CFA vs. CXB (CFA)	5.4	4	1.4	0.8923	5	5	2.219	200
256	CFA vs. DF-NE (CFA)	5.4	4.6	0.8	0.8923	5	5	1.268	200
257	CXB (CFA) vs. DF-NE (CFA)	4	4.6	-0.6	0.8923	5	5	0.951	200

	A	B	C	D	E	F	G	H	I
1	**Von Frey ASSAY in FEMALE MICE**								
2									
3	**Two-way ANOVA**	**Ordinary**							
4	**Alpha**	**0.05**							
5									
6	**Source of Variation**	**% of total varia**	**P value**	**P value summa**	**Significant?**				
7	**Interaction**	**11.46**	**< 0.0001**	********	**Yes**				
8	**Row Factor**	**44.57**	**< 0.0001**	********	**Yes**				
9	**Column Factor**	**27.63**	**< 0.0001**	********	**Yes**				
10									
11	**ANOVA table**	**SS**	**DF**	**MS**	**F (DFn, DFd)**	**P value**			
12	**Interaction**	**247.3**	**36**	**6.87**	**F (36, 200) = 3.**	**P < 0.0001**			
13	**Row Factor**	**962.3**	**9**	**106.9**	**F (9, 200) = 60.**	**P < 0.0001**			
14	**Column Factor**	**596.5**	**4**	**149.1**	**F (4, 200) = 84.**	**P < 0.0001**			
15	**Residual**	**352.8**	**200**	**1.764**					
16									
17	**Number of missing values**	**0**							
18									
19	Within each row, compare columns (simple effects within rows)								
20									
21	Number of families	10							
22	Number of comparisons per family	10							
23	Alpha	0.05							
24									
25	**Tukey's multiple comparisons test**	Mean Diff.	95% CI of diff.	Significant?	Summary				
26									
27	**Day 1**								
28	Saline vs. CXB-NE (CFA)	-1.6	-3.913 to 0.7125	No	ns				
29	Saline vs. CFA	-5.9	-8.213 to -3.587	Yes	****				
30	Saline vs. CXB (CFA)	-4.2	-6.513 to -1.887	Yes	****				
31	Saline vs. DF-NE (CFA)	-6	-8.313 to -3.687	Yes	****				
32	CXB-NE (CFA) vs. CFA	-4.3	-6.613 to -1.987	Yes	****				
33	**CXB-NE (CFA) vs. CXB (CFA)**	**-2.6**	**-4.913 to -0.2875**	**Yes**	*****				
34	**CXB-NE (CFA) vs. DF-NE (CFA)**	**-4.4**	**-6.713 to -2.087**	**Yes**	********				
35	CFA vs. CXB (CFA)	1.7	-0.6125 to 4.013	No	ns				
36	CFA vs. DF-NE (CFA)	-0.1	-2.413 to 2.213	No	ns				
37	CXB (CFA) vs. DF-NE (CFA)	-1.8	-4.113 to 0.5125	No	ns				
38									
39	**Day 3**								
40	Saline vs. CXB-NE (CFA)	-1.4	-3.713 to 0.9125	No	ns				
41	Saline vs. CFA	-5	-7.313 to -2.687	Yes	****				
42	Saline vs. CXB (CFA)	-5.2	-7.513 to -2.887	Yes	****				
43	Saline vs. DF-NE (CFA)	-5	-7.313 to -2.687	Yes	****				
44	**CXB-NE (CFA) vs. CFA**	**-3.6**	**-5.913 to -1.287**	**Yes**	*******				
45	**CXB-NE (CFA) vs. CXB (CFA)**	**-3.8**	**-6.113 to -1.487**	**Yes**	*******				
46	**CXB-NE (CFA) vs. DF-NE (CFA)**	**-3.6**	**-5.913 to -1.287**	**Yes**	*******				
47	CFA vs. CXB (CFA)	-0.2	-2.513 to 2.113	No	ns				
48	CFA vs. DF-NE (CFA)	0	-2.313 to 2.313	No	ns				
49	CXB (CFA) vs. DF-NE (CFA)	0.2	-2.113 to 2.513	No	ns				
50									
51	**Day 6**								
52	Saline vs. CXB-NE (CFA)	-3.3	-5.613 to -0.9875	Yes	**				
53	Saline vs. CFA	-5.9	-8.213 to -3.587	Yes	****				
54	Saline vs. CXB (CFA)	-6.5	-8.813 to -4.187	Yes	****				
55	Saline vs. DF-NE (CFA)	-6.9	-9.213 to -4.587	Yes	****				
56	**CXB-NE (CFA) vs. CFA**	**-2.6**	**-4.913 to -0.2875**	**Yes**	*****				
57	**CXB-NE (CFA) vs. CXB (CFA)**	**-3.2**	**-5.513 to -0.8875**	**Yes**	******				
58	**CXB-NE (CFA) vs. DF-NE (CFA)**	**-3.6**	**-5.913 to -1.287**	**Yes**	*******				
59	CFA vs. CXB (CFA)	-0.6	-2.913 to 1.713	No	ns				
60	CFA vs. DF-NE (CFA)	-1	-3.313 to 1.313	No	ns				
61	CXB (CFA) vs. DF-NE (CFA)	-0.4	-2.713 to 1.913	No	ns				
62									
63	**Day 11**								
64	Saline vs. CXB-NE (CFA)	-2.4	-4.713 to -0.08750	Yes	*				
65	Saline vs. CFA	-4.8	-7.113 to -2.487	Yes	****				
66	Saline vs. CXB (CFA)	-5.2	-7.513 to -2.887	Yes	****				
67	Saline vs. DF-NE (CFA)	-4.6	-6.913 to -2.287	Yes	****				
68	**CXB-NE (CFA) vs. CFA**	**-2.4**	**-4.713 to -0.08750**	**Yes**	*****				
69	**CXB-NE (CFA) vs. CXB (CFA)**	**-2.8**	**-5.113 to -0.4875**	**Yes**	******				
70	**CXB-NE (CFA) vs. DF-NE (CFA)**	**-2.2**	**-4.513 to 0.1125**	**No**	**ns**				
71	CFA vs. CXB (CFA)	-0.4	-2.713 to 1.913	No	ns				
72	CFA vs. DF-NE (CFA)	0.2	-2.113 to 2.513	No	ns				
73	CXB (CFA) vs. DF-NE (CFA)	0.6	-1.713 to 2.913	No	ns				
74									
75	**Day 12**								
76	Saline vs. CXB-NE (CFA)	-3.6	-5.913 to -1.287	Yes	***				
77	Saline vs. CFA	-4.2	-6.513 to -1.887	Yes	****				
78	Saline vs. CXB (CFA)	-4.2	-6.513 to -1.887	Yes	****				
79	Saline vs. DF-NE (CFA)	-5.6	-7.913 to -3.287	Yes	****				
80	**CXB-NE (CFA) vs. CFA**	**-0.6**	**-2.913 to 1.713**	**No**	**ns**				
81	**CXB-NE (CFA) vs. CXB (CFA)**	**-0.6**	**-2.913 to 1.713**	**No**	**ns**				
82	**CXB-NE (CFA) vs. DF-NE (CFA)**	**-2**	**-4.313 to 0.3125**	**No**	**ns**				
83	CFA vs. CXB (CFA)	0	-2.313 to 2.313	No	ns				
84	CFA vs. DF-NE (CFA)	-1.4	-3.713 to 0.9125	No	ns				
85	CXB (CFA) vs. DF-NE (CFA)	-1.4	-3.713 to 0.9125	No	ns				
86									
87	18								
88	Saline vs. CXB-NE (CFA)	-4.2	-6.513 to -1.887	Yes	****				
89	Saline vs. CFA	-5	-7.313 to -2.687	Yes	****				
90	Saline vs. CXB (CFA)	-4.8	-7.113 to -2.487	Yes	****				
91	Saline vs. DF-NE (CFA)	-3.4	-5.713 to -1.087	Yes	***				
92	CXB-NE (CFA) vs. CFA	-0.8	-3.113 to 1.513	No	ns				

	A	B	C	D	E	F	G	H	I
93	CXB-NE (CFA) vs. CXB (CFA)	-0.6	-2.913 to 1.713	No	ns				
94	CXB-NE (CFA) vs. DF-NE (CFA)	0.8	-1.513 to 3.113	No	ns				
95	CFA vs. CXB (CFA)	0.2	-2.113 to 2.513	No	ns				
96	CFA vs. DF-NE (CFA)	1.6	-0.7125 to 3.913	No	ns				
97	CXB (CFA) vs. DF-NE (CFA)	1.4	-0.9125 to 3.713	No	ns				
98									
99	24								
100	Saline vs. CXB-NE (CFA)	-4.1	-6.413 to -1.787	Yes	****				
101	Saline vs. CFA	-4.7	-7.013 to -2.387	Yes	****				
102	Saline vs. CXB (CFA)	-2.8	-5.113 to -0.4875	Yes	**				
103	Saline vs. DF-NE (CFA)	-3.7	-6.013 to -1.387	Yes	***				
104	CXB-NE (CFA) vs. CFA	-0.6	-2.913 to 1.713	No	ns				
105	CXB-NE (CFA) vs. CXB (CFA)	1.3	-1.013 to 3.613	No	ns				
106	CXB-NE (CFA) vs. DF-NE (CFA)	0.4	-1.913 to 2.713	No	ns				
107	CFA vs. CXB (CFA)	1.9	-0.4125 to 4.213	No	ns				
108	CFA vs. DF-NE (CFA)	1	-1.313 to 3.313	No	ns				
109	CXB (CFA) vs. DF-NE (CFA)	-0.9	-3.213 to 1.413	No	ns				
110									
111	32								
112	Saline vs. CXB-NE (CFA)	-3.6	-5.913 to -1.287	Yes	***				
113	Saline vs. CFA	-3.3	-5.613 to -0.9875	Yes	**				
114	Saline vs. CXB (CFA)	-1.3	-3.613 to 1.013	No	ns				
115	Saline vs. DF-NE (CFA)	-2.6	-4.913 to -0.2875	Yes	*				
116	CXB-NE (CFA) vs. CFA	0.3	-2.013 to 2.613	No	ns				
117	CXB-NE (CFA) vs. CXB (CFA)	2.3	-0.01250 to 4.613	No	ns				
118	CXB-NE (CFA) vs. DF-NE (CFA)	1	-1.313 to 3.313	No	ns				
119	CFA vs. CXB (CFA)	2	-0.3125 to 4.313	No	ns				
120	CFA vs. DF-NE (CFA)	0.7	-1.613 to 3.013	No	ns				
121	CXB (CFA) vs. DF-NE (CFA)	-1.3	-3.613 to 1.013	No	ns				
122									
123	40								
124	Saline vs. CXB-NE (CFA)	-2.5	-4.813 to -0.1875	Yes	*				
125	Saline vs. CFA	-3.6	-5.913 to -1.287	Yes	***				
126	Saline vs. CXB (CFA)	-1.6	-3.913 to 0.7125	No	ns				
127	Saline vs. DF-NE (CFA)	-2.2	-4.513 to 0.1125	No	ns				
128	CXB-NE (CFA) vs. CFA	-1.1	-3.413 to 1.213	No	ns				
129	CXB-NE (CFA) vs. CXB (CFA)	0.9	-1.413 to 3.213	No	ns				
130	CXB-NE (CFA) vs. DF-NE (CFA)	0.3	-2.013 to 2.613	No	ns				
131	CFA vs. CXB (CFA)	2	-0.3125 to 4.313	No	ns				
132	CFA vs. DF-NE (CFA)	1.4	-0.9125 to 3.713	No	ns				
133	CXB (CFA) vs. DF-NE (CFA)	-0.6	-2.913 to 1.713	No	ns				
134									
135									
136	Test details	Mean 1	Mean 2	Mean Diff.	SE of diff.	N1	N2	q	DF
137									
138									
139	1								
140	Saline vs. CXB-NE (CFA)	1.8	3.4	-1.6	0.84	5	5	2.694	200
141	Saline vs. CFA	1.8	7.7	-5.9	0.84	5	5	9.933	200
142	Saline vs. CXB (CFA)	1.8	6	-4.2	0.84	5	5	7.071	200
143	Saline vs. DF-NE (CFA)	1.8	7.8	-6	0.84	5	5	10.1	200
144	CXB-NE (CFA) vs. CFA	3.4	7.7	-4.3	0.84	5	5	7.239	200
145	CXB-NE (CFA) vs. CXB (CFA)	3.4	6	-2.6	0.84	5	5	4.377	200
146	CXB-NE (CFA) vs. DF-NE (CFA)	3.4	7.8	-4.4	0.84	5	5	7.407	200
147	CFA vs. CXB (CFA)	7.7	6	1.7	0.84	5	5	2.862	200
148	CFA vs. DF-NE (CFA)	7.7	7.8	-0.1	0.84	5	5	0.1683	200
149	CXB (CFA) vs. DF-NE (CFA)	6	7.8	-1.8	0.84	5	5	3.03	200
150									
151	3								
152	Saline vs. CXB-NE (CFA)	3.8	5.2	-1.4	0.84	5	5	2.357	200
153	Saline vs. CFA	3.8	8.8	-5	0.84	5	5	8.417	200
154	Saline vs. CXB (CFA)	3.8	9	-5.2	0.84	5	5	8.754	200
155	Saline vs. DF-NE (CFA)	3.8	8.8	-5	0.84	5	5	8.417	200
156	CXB-NE (CFA) vs. CFA	5.2	8.8	-3.6	0.84	5	5	6.061	200
157	CXB-NE (CFA) vs. CXB (CFA)	5.2	9	-3.8	0.84	5	5	6.397	200
158	CXB-NE (CFA) vs. DF-NE (CFA)	5.2	8.8	-3.6	0.84	5	5	6.061	200
159	CFA vs. CXB (CFA)	8.8	9	-0.2	0.84	5	5	0.3367	200
160	CFA vs. DF-NE (CFA)	8.8	8.8	0	0.84	5	5	0	200
161	CXB (CFA) vs. DF-NE (CFA)	9	8.8	0.2	0.84	5	5	0.3367	200
162									
163	6								
164	Saline vs. CXB-NE (CFA)	2.3	5.6	-3.3	0.84	5	5	5.556	200
165	Saline vs. CFA	2.3	8.2	-5.9	0.84	5	5	9.933	200
166	Saline vs. CXB (CFA)	2.3	8.8	-6.5	0.84	5	5	10.94	200
167	Saline vs. DF-NE (CFA)	2.3	9.2	-6.9	0.84	5	5	11.62	200
168	CXB-NE (CFA) vs. CFA	5.6	8.2	-2.6	0.84	5	5	4.377	200
169	CXB-NE (CFA) vs. CXB (CFA)	5.6	8.8	-3.2	0.84	5	5	5.387	200
170	CXB-NE (CFA) vs. DF-NE (CFA)	5.6	9.2	-3.6	0.84	5	5	6.061	200
171	CFA vs. CXB (CFA)	8.2	8.8	-0.6	0.84	5	5	1.01	200
172	CFA vs. DF-NE (CFA)	8.2	9.2	-1	0.84	5	5	1.683	200
173	CXB (CFA) vs. DF-NE (CFA)	8.8	9.2	-0.4	0.84	5	5	0.6734	200
174									
175	11								
176	Saline vs. CXB-NE (CFA)	2	4.4	-2.4	0.84	5	5	4.04	200
177	Saline vs. CFA	2	6.8	-4.8	0.84	5	5	8.081	200
178	Saline vs. CXB (CFA)	2	7.2	-5.2	0.84	5	5	8.754	200
179	Saline vs. DF-NE (CFA)	2	6.6	-4.6	0.84	5	5	7.744	200
180	CXB-NE (CFA) vs. CFA	4.4	6.8	-2.4	0.84	5	5	4.04	200
181	CXB-NE (CFA) vs. CXB (CFA)	4.4	7.2	-2.8	0.84	5	5	4.714	200
182	CXB-NE (CFA) vs. DF-NE (CFA)	4.4	6.6	-2.2	0.84	5	5	3.704	200
183	CFA vs. CXB (CFA)	6.8	7.2	-0.4	0.84	5	5	0.6734	200
184	CFA vs. DF-NE (CFA)	6.8	6.6	0.2	0.84	5	5	0.3367	200

	A	B	C	D	E	F	G	H	I
185	CXB (CFA) vs. DF-NE (CFA)	7.2	6.6	0.6	0.84	5	5	1.01	200
186									
187	12								
188	Saline vs. CXB-NE (CFA)	2.6	6.2	-3.6	0.84	5	5	6.061	200
189	Saline vs. CFA	2.6	6.8	-4.2	0.84	5	5	7.071	200
190	Saline vs. CXB (CFA)	2.6	6.8	-4.2	0.84	5	5	7.071	200
191	Saline vs. DF-NE (CFA)	2.6	8.2	-5.6	0.84	5	5	9.428	200
192	CXB-NE (CFA) vs. CFA	6.2	6.8	-0.6	0.84	5	5	1.01	200
193	CXB-NE (CFA) vs. CXB (CFA)	6.2	6.8	-0.6	0.84	5	5	1.01	200
194	CXB-NE (CFA) vs. DF-NE (CFA)	6.2	8.2	-2	0.84	5	5	3.367	200
195	CFA vs. CXB (CFA)	6.8	6.8	0	0.84	5	5	0	200
196	CFA vs. DF-NE (CFA)	6.8	8.2	-1.4	0.84	5	5	2.357	200
197	CXB (CFA) vs. DF-NE (CFA)	6.8	8.2	-1.4	0.84	5	5	2.357	200
198									
199	18								
200	Saline vs. CXB-NE (CFA)	3.4	7.6	-4.2	0.84	5	5	7.071	200
201	Saline vs. CFA	3.4	8.4	-5	0.84	5	5	8.417	200
202	Saline vs. CXB (CFA)	3.4	8.2	-4.8	0.84	5	5	8.081	200
203	Saline vs. DF-NE (CFA)	3.4	6.8	-3.4	0.84	5	5	5.724	200
204	CXB-NE (CFA) vs. CFA	7.6	8.4	-0.8	0.84	5	5	1.347	200
205	CXB-NE (CFA) vs. CXB (CFA)	7.6	8.2	-0.6	0.84	5	5	1.01	200
206	CXB-NE (CFA) vs. DF-NE (CFA)	7.6	6.8	0.8	0.84	5	5	1.347	200
207	CFA vs. CXB (CFA)	8.4	8.2	0.2	0.84	5	5	0.3367	200
208	CFA vs. DF-NE (CFA)	8.4	6.8	1.6	0.84	5	5	2.694	200
209	CXB (CFA) vs. DF-NE (CFA)	8.2	6.8	1.4	0.84	5	5	2.357	200
210									
211	24								
212	Saline vs. CXB-NE (CFA)	3.5	7.6	-4.1	0.84	5	5	6.902	200
213	Saline vs. CFA	3.5	8.2	-4.7	0.84	5	5	7.912	200
214	Saline vs. CXB (CFA)	3.5	6.3	-2.8	0.84	5	5	4.714	200
215	Saline vs. DF-NE (CFA)	3.5	7.2	-3.7	0.84	5	5	6.229	200
216	CXB-NE (CFA) vs. CFA	7.6	8.2	-0.6	0.84	5	5	1.01	200
217	CXB-NE (CFA) vs. CXB (CFA)	7.6	6.3	1.3	0.84	5	5	2.189	200
218	CXB-NE (CFA) vs. DF-NE (CFA)	7.6	7.2	0.4	0.84	5	5	0.6734	200
219	CFA vs. CXB (CFA)	8.2	6.3	1.9	0.84	5	5	3.199	200
220	CFA vs. DF-NE (CFA)	8.2	7.2	1	0.84	5	5	1.683	200
221	CXB (CFA) vs. DF-NE (CFA)	6.3	7.2	-0.9	0.84	5	5	1.515	200
222									
223	32								
224	Saline vs. CXB-NE (CFA)	3.7	7.3	-3.6	0.84	5	5	6.061	200
225	Saline vs. CFA	3.7	7	-3.3	0.84	5	5	5.556	200
226	Saline vs. CXB (CFA)	3.7	5	-1.3	0.84	5	5	2.189	200
227	Saline vs. DF-NE (CFA)	3.7	6.3	-2.6	0.84	5	5	4.377	200
228	CXB-NE (CFA) vs. CFA	7.3	7	0.3	0.84	5	5	0.505	200
229	CXB-NE (CFA) vs. CXB (CFA)	7.3	5	2.3	0.84	5	5	3.872	200
230	CXB-NE (CFA) vs. DF-NE (CFA)	7.3	6.3	1	0.84	5	5	1.683	200
231	CFA vs. CXB (CFA)	7	5	2	0.84	5	5	3.367	200
232	CFA vs. DF-NE (CFA)	7	6.3	0.7	0.84	5	5	1.178	200
233	CXB (CFA) vs. DF-NE (CFA)	5	6.3	-1.3	0.84	5	5	2.189	200
234									
235	40								
236	Saline vs. CXB-NE (CFA)	4.2	6.7	-2.5	0.84	5	5	4.209	200
237	Saline vs. CFA	4.2	7.8	-3.6	0.84	5	5	6.061	200
238	Saline vs. CXB (CFA)	4.2	5.8	-1.6	0.84	5	5	2.694	200
239	Saline vs. DF-NE (CFA)	4.2	6.4	-2.2	0.84	5	5	3.704	200
240	CXB-NE (CFA) vs. CFA	6.7	7.8	-1.1	0.84	5	5	1.852	200
241	CXB-NE (CFA) vs. CXB (CFA)	6.7	5.8	0.9	0.84	5	5	1.515	200
242	CXB-NE (CFA) vs. DF-NE (CFA)	6.7	6.4	0.3	0.84	5	5	0.505	200
243	CFA vs. CXB (CFA)	7.8	5.8	2	0.84	5	5	3.367	200
244	CFA vs. DF-NE (CFA)	7.8	6.4	1.4	0.84	5	5	2.357	200
245	CXB (CFA) vs. DF-NE (CFA)	5.8	6.4	-0.6	0.84	5	5	1.01	200
